# Identification and validation of a novel cuproptosis-related lncRNA gene signature to predict prognosis and immune response in bladder cancer

**DOI:** 10.1007/s12672-022-00596-w

**Published:** 2022-12-01

**Authors:** Jia Chen, Yu Guan, Chun Li, Hexi Du, Chaozhao Liang

**Affiliations:** 1 Department of Urology, The First Affiliated Hospital of Anhui Medical University, 218th Jixi Road, Shushan District, Hefei, 230022 Anhui People’s Republic of China; 2 Institute of Urology, Anhui Medical University, 218th Jixi Road, Shushan District, Hefei, 230022 Anhui People’s Republic of China; 3 Anhui Province Key Laboratory of Genitourinary Diseases, 218th Jixi Road, Shushan District, Hefei, 230022 Anhui People’s Republic of China; 4 Department of General Surgery, The First Affiliated Hospital of Anhui Medical University, 218th Jixi Road, Hefei, 230022 Anhui People’s Republic of China

**Keywords:** Bladder cancer, Cuproptosis, LncRNA, Prognosis, Immune response

## Abstract

**Purpose:**

Bladder cancer (BCa) is one of the most common malignant tumors in the urogenital system, characterized by the high recurrence rate, mortality rate and poor prognosis. Based on cuproptosis-related long noncoding RNAs (CRLs), this study set out to create a prediction signature to evaluate the prognosis of patients with BCa.

**Methods:**

RNA-seq data including CRLs and related clinicopathological data were gathered from The Cancer Genome Atlas (TCGA) database (n = 428). The predictive signature was constructed after correlation analysis. Subsequently, relying on the analyzed data from the TCGA database and our sample collection, we examined and verified the connections between CRLs model and important indexes included prognosis, route and functional enrichment, tumor immune evasion, tumor mutation, and treatment sensitivity.

**Results:**

Patients in the high-risk group had lower overall survival (OS) than that of low-risk group. Compared with clinicopathological variables, CRLs features have better predictive value according to receiver operating characteristic (ROC) curve. The expression level of CRLs was highly associated with the tumor progress, tumor microenvironment and tumor immune escape. Additionally, we identified that the mutation of TP53, TTN, KMT2D and MUC16 gene were founded in patients with BCa. Lapatinib, pazopanib, saracatinib, gemcitabine, paclitaxel and palenolactone had good antitumor effects for BCa patients in the high-risk group (all P < 0.001).

**Conclusion:**

This study revealed the effects of CRLs on BCa and further established CRLs model, which can be used in clinic for predicting prognosis, immunological response and treatment sensitivity inpatient with BCa.

**Supplementary Information:**

The online version contains supplementary material available at 10.1007/s12672-022-00596-w.

## Introduction

Although bladder cancer (BCa) patients have received surgery, chemotherapy, radiotherapy, and other treatments, the number of BCa cases has been on the rise worldwide in the past 20 years. The risk of recurrence, progression and metastasis is high, especially in patients resistant to adjuvant or neoadjuvant therapy [1, 2]. Therefore, reliable prognostic biomarkers have a guiding role in clinical treatment, and immunotargeted therapy may be a feasible way to improve the prognosis of BCa. Recently, cell death has become a research hotspot, such as apoptosis [3], pyroptosis [4–6], autophagy and ferroptosis [7], etc., which are highly associated with the tumors. The inflammatory infiltration around the tumor may be associated with overall survival. The Cancer Genome Atlas (TCGA) study found that the gene mutation rate of chromatin remodeling in bladder cancer is higher than that in other tumors, which may become a new therapeutic target [8]. Pembrolizumab was recently approved by the FDA for the treatment of untreated, unresectable tumors with a high mutational burden (TMB) [9, 10].

Recently, a new type of cell death induced by intracellular copper(Cu), called cuproptosis, has been discovered. It differs from the previously found pattern of cell death, with a recent study showing that intracellular Cu induces a new form of regulated cell death. It is well known that Cu is a human trace metal in cells and plays an indispensable role in maintaining protein function. Excessive Cu can also cause cytotoxicity, but the exact mechanism is unclear [11, 12]. Increasing evidence suggests that disruption of Cu homeostasis may be associated with tumor growth and death, and tumor immunity [13]. It has been shown that cuproptosis regulates TME through cells such as CD8 + T, thereby promoting tumor growth and progression [14]. Therefore, cuproptosis has an essential relationship with the occurrence and development of tumor.

LncRNA is a class of heterogeneous transcripts with at least 200 bases and has a limited affinity for protein coding [15–17]. LncRNAs affect a variety of human diseases, including tumorigenesis, and they are important regulatory factors of tumor diseases by mediating the behavior of tumor cells [18, 19]. LncRNAs are emerging regulators involved in gene expression and various physiological and pathological processes. Some scholars believe that abnormal lncRNAs in bladder cancer are closely related to the development and prognosis of the disease, and increasing evidence shows that lncRNAs have the potential to regulate ferroptosis, apoptosis and autophagy in bladder cancer, and play a complex and precise regulatory role in cancer initiation [20–24]. They not only regulate the proliferation, differentiation, invasion and metastasis of cancer cells but also regulate the metabolic reprogramming of cancer cells [25].

In our study, we constructed a CRLs model to predict the prognosis of BCa, which may be associated with immune infiltration, and provide guidance for selecting targeted or immune-boosting therapy drugs in patients with BCa.

## Materials and methods

### Datasets

**Fig. 1:**
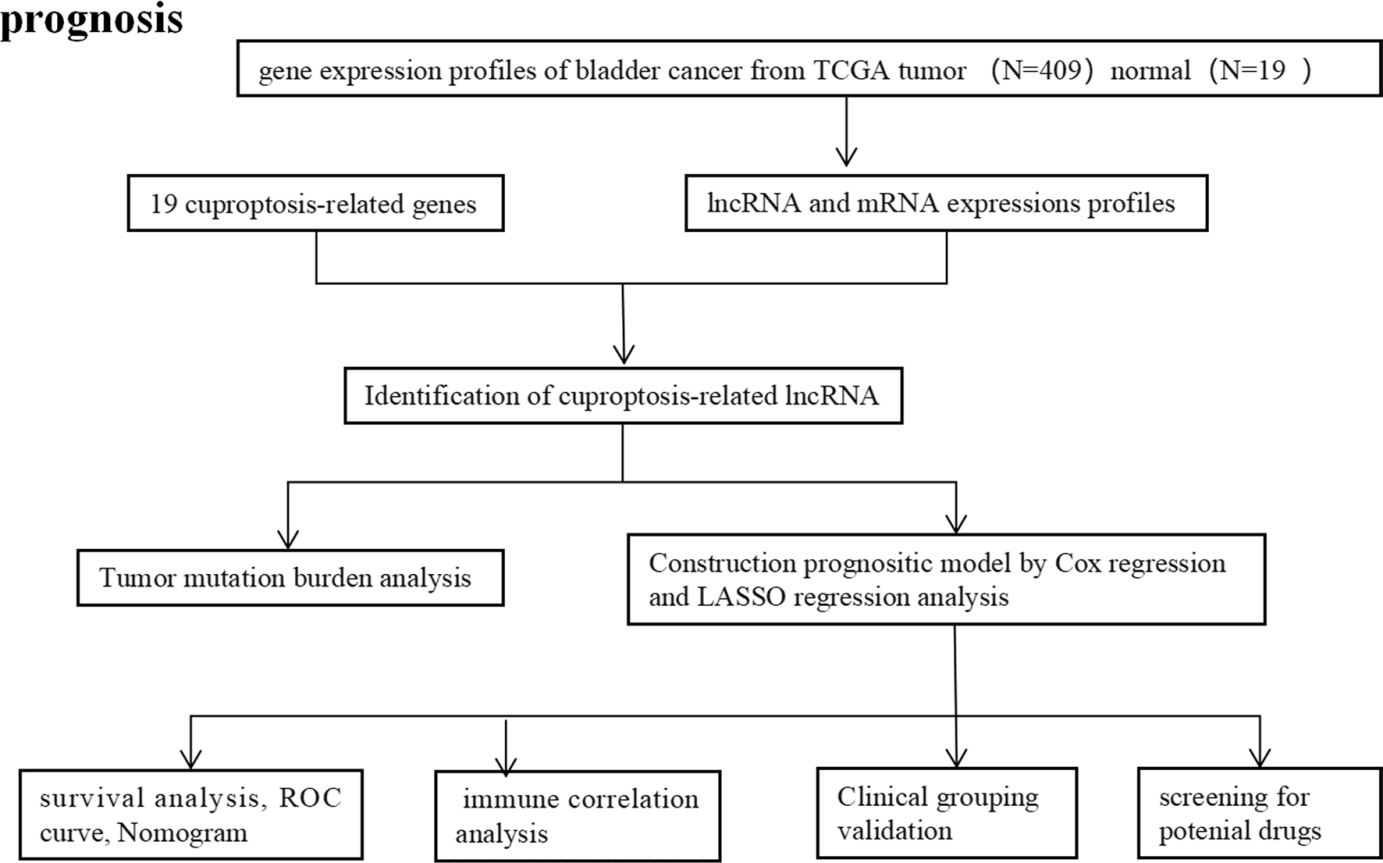
The flowchart of the overall procedures. This flowchart illustrates the process of data collection and analysis for prognostic study

The design process and grouping are illustrated in Fig. 1. From the TCGA database (https://portal.gdc.cancer.gov/repository) to download data, specimens of 428 cases, in which the BCa 409 cases, normal bladder tissue 19 cases. The RNA sequencing (including mRNA and lncRNA) data of BCa patient samples, normal samples, clinical information and tumor mutation data. Differentially expressed genes (DEGs) were identified between BCa and normal bladder tissues.

### Identification of the cuproptosis-related lncRNAs in the TCGA cohort

We obtained 19 cuproptosis-related genes (Table S2) from previous literatures [10, 11] and identified CRLs. We used the "Limma" R package to detect the correlation between cuproptosis-related gene expression and lncRNAs expression. CorFilter = 0.4 and p-value Filter = 0.001 were used as screening conditions to obtain the expression of CRLs.

### Establishment and validation of the CRLs model of prognosis

Overall survival was analyzed by univariate cox regression and least absolute shrinkage and selection operator (LASSO) to screen prognostic value in cuproptosis-related lncRNAs. Four hundred and five samples were left in the final cohort for analysis, with 4 unknown clinical samples excluded. They were randomly assigned to the train group and the test group. Subsequently, we used the R package ‘‘glmnet’’ to identify the genetic signatures containing the most valuable biomarkers for prognosis and to calculate the risk score for each sample in all data sets based on the target gene volume. A risk scoring formula was then established based on the linear combination of the prognostic risk score of lncRNA with the gene expression level multiplied regression model as follows: BCa prognosis risk score = $\sum_{(n=1)}^{i}Coefi*Xi$ expression level ($Coefi$ indicates the coefficients, $Xi$ represents the standardized level of gene expression) (coef*lncRNA1 exp.) + (coef*lncRNA2 exp.) + (coef*lncRNA3 exp.) ……+ (coef*lncRNAn exp.). In the survival analysis, samples were divided into high-risk groups and low-risk groups according to the optimal cutoff value of risk score analyzed by R package ‘‘survminer’’. The correlation between lncRNA and cuproptosis-related genes was compared using ‘‘ggplot2’’ method, and the heat map was drawn. Afterwards, Kaplan–Meier analysis was used to investigate the prognostic significance of a 9-CRLs signature in BCa, and ‘‘pheatmap’’ R package was used to divide patients into low and high-risk groups based on risk scores and plotted a risk curve. Then, we performed multivariate cox regression analyses to evaluate whether the risk score model had an excellent prognosis, independent of other clinical information, such as age, gender, grade, and stage. Then, according to these clinical shapes, all tumor patients were randomly divided into ‘‘train’’ and ‘‘test’’ groups, and the risk curves were drawn respectively for comparison.

Moreover, we assessed the predictive power of this risk classification score by analyzing the operating characteristics of subject survival (ROC) using the ‘‘timeROC’’ R package in all data sets. Subsequently, we again used the Kaplan–Meier model to analyze the prognostic significance of clinical features. The ‘‘Scatterplot3D’’ R package was used to plot PCA components of all genes, cuproptosis-related genes, cuproptosis-related lncRNA and lncRNA models. Besides, a nomogram for prognostic prediction was constructed on the ground of the TCGA dataset. Finally, all clinical parameters with independent prognosis were included in the TCGA dataset, and a nomogram was constructed to predict the OS of BCa patients at 1, 3, and 5 years by a stepwise cox regression model.

### RNA extraction and quantitative real-time analysis (qRT-PCR)

Total RNA was isolated from human bladder tissues using TRIzol reagent (Invitrogen, USA).We reverse transcribed the extracted total RNA into stable cDNA using the PrimeScript RT Master Mix kit (Takara, USA), followed by qRT-PCR using SYBR qPCR Master Mix (Vazyme, China). Finally, ΔΔCT of each sample was calculated and normalized to GAPDH. Primer sequences of lncRNAs are shown in Table 1.

**Table 1:** primer sequences used in the qRT-PCR assay

Primer Name	Sequence(5′–3′)
PCAT7(For)	TAGGCCAGAAGGTTCCCAAG
PCAT7(Rev)	GCAACACCTCTGCTATGTGG
SLC12A2-DT(For)	CTGATGCGGTAGTGAGGCTA
SLC12A2-DT(Rev)	CCCGTGATAATTGCGTGGAT
MPP7-DT(For)	AGTAGGAGCCATCCCAGAGA
MPP7-DT(Rev)	CCATCCAGGCTAGGCAATCT
FAM87B(For)	AGCCAGTGAGGCAAGTGTAT
FAM87B(Rev)	TTCCATTCCGTTCCATTGGC
UBE2Q1-AS1(For)	AAGCCTACCTCAGGCATCTC
UBE2Q1-AS1(Rev)	TGCATTCTAGCAAGCCACTG
GAPDH(For)	GGAGCGAGATCCCTCCAAAAT
GAPDH(Rev)	GGCTGTTGTCATACTTCTCATGG

### GO and KEGG analysis of the differential expression cuproptosis-related lncRNAs (DE-CRLs)

BCa patients obtained from the TCGA cohort were classified into a low-risk group and a high-risk group on basis of their median risk score. According to the specific standard |log2FC|≥ 1 and P value < 0.05 were regarded as the specific standard, we then extracted the DE-CRLs between the low-risk and high-risk groups. Afterwards, we applied to enriched GO and KEGG pathways by application of the "cluster profiler" package, and the "GO plot, ggplt2 and circlize" package was used to visualize the results. P-value and q-value/FDR < 0.05 were considered statistically significant.

### Tumor mutation burden difference and survival analysis

We downloaded tumor mutation data from TCGA database and divided patients into high-risk and low-risk groups by calculating tumor mutation burden. The waterfall diagram was drawn using ‘‘maftools’’ R package (only 15 genes with the highest mutation frequency were displayed). Then, the survival analysis of the high-low mutation burden group and the survival curve was calculated using the "survminer" R package.

### Immune correlation analysis and immune escape

‘‘GSVA’’ and ‘‘GSEABase’’ R packages were performed to identify the potential molecular mechanisms or functional pathways with gene set files for immune functions that involve the CRLs signature. We downloaded the tumor escape data from the TIDE website (http://tide.dfci.harvard.edu/) and used ‘‘ggpubr’’ R package for data processing and visualization.

### Screening for potential drugs and susceptibility to bladder cancer

To evaluate clinical drug efficacy in BCa, we classified BCa patients from the TCGA database and calculated the IC50 (IC50 is 50% inhibition concentration, that is, the corresponding concentration when B/B0 = 50%. 50% inhibition is used to measure the sensitivity of the antibody. The lower the 50% inhibition, the higher the sensitivity of the antibody) of commonly used chemotherapeutic agents using the algorithm [26, 27] and the corresponding ‘‘Predictive’’ R package. The algorithm allows users to predict clinical chemotherapy responses by creating statistical models from gene expression and drug sensitivity data from cancer genome project cell lines, using only baseline tumor gene expression data.

### Statistical analysis

All statistical analyses were performed using R software (v4.1.3). P-value < 0.05 was regarded as statistically significant.

## Results

### Identification of differentially expressed CRLs

**Fig. 2:**
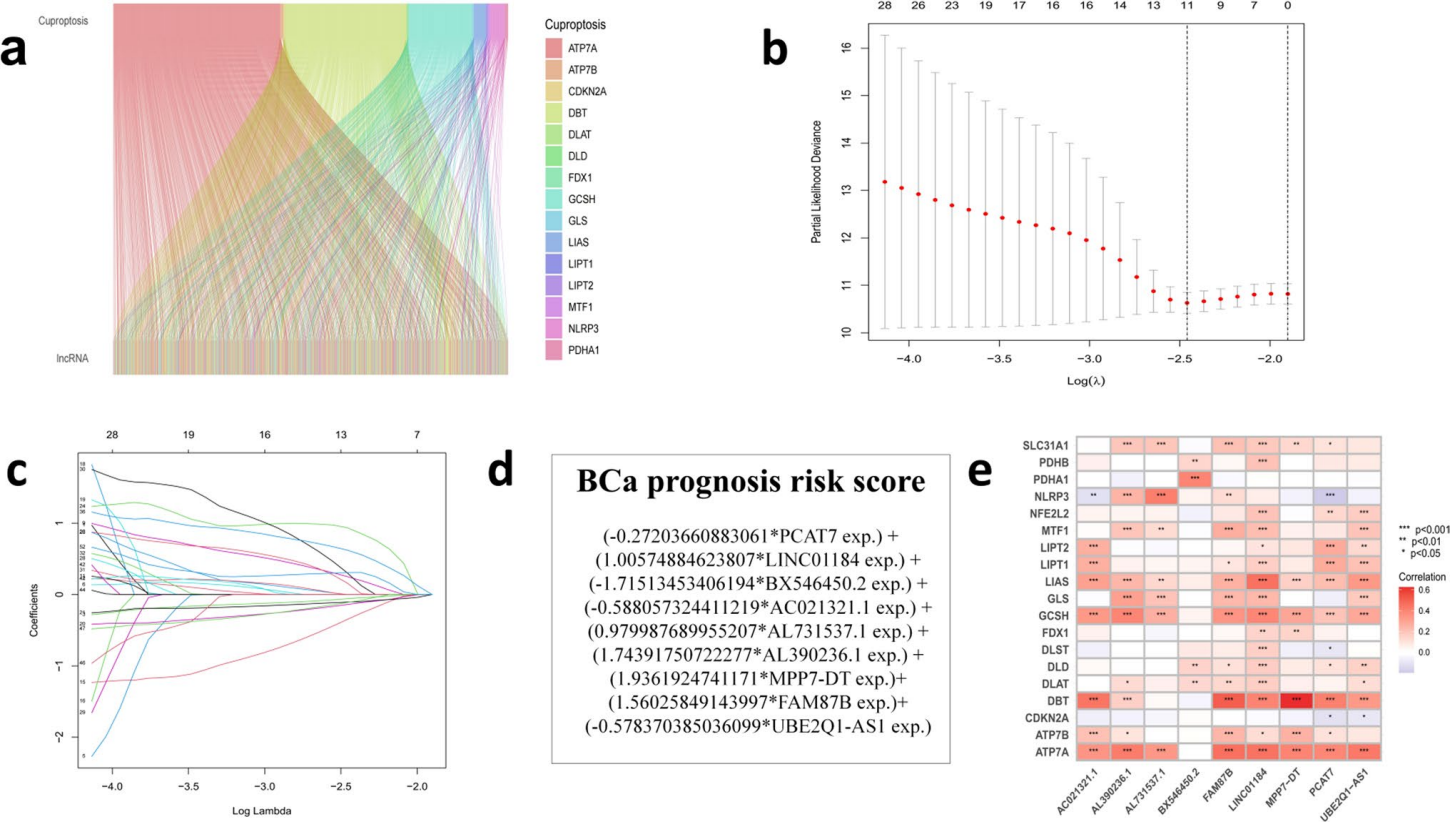
A screen of differentially expressed cuproptosis-related lncRNAs In bladder cancer **a** Co-expression analysis results show the correlation between cuprotosis-related genes and lncRNAs. **b**,**c** LASSO cox regression for the prognostic value of the cuprotosis-related lncRNAs. **d** CRLs risk model formula. **e** The heat map indicated a correlation between the 19 cuproptosis-related genes and 9 signature lncRNAs

428 bladder cancer tissues, including 409 cancer samples and 19 normal samples, were obtained from the TCGA database, which were divided into mRNAs and lncRNAs. Then, 19 cuproptosis-related genes obtained from the literatures were co-expressed with lncRNAs obtained from the database, and the results were compared, correlated and analyzed to acquire CRLs (Fig. 2a). In addition, CRLs were associated with the survival time of patients, and all cases were branched into the train group and the test group randomly. The results showed that the clinical characteristics of the two groups were basically consistent, suggesting that a comparative analysis between groups can be conducted (Table 2). Then, 14 CRLs were obtained by univariate cox regression and Lasso regression analysis (Fig. 2b, c), and 9 CRLs (PCAT7, LINC01184 (SLC12A2-DT), BX546450.2, AC021321.1, AL731537.1, AL390236.1, MPP7-DT, FAM87B, UBE2Q1-AS1) models were obtained by multivariate cox regression analysis. Then, we first used the train group to build the model formula: BCa prognosis risk score = (− 0.27203660883061*PCAT7 exp.) + (1.00574884623807*LINC01184 exp.) + (-1.71513453406194*BX546450.2 exp.) + (− 0.588057324411219*AC021321.1 exp.) + (0.979987689955207*AL731537.1 exp.) + (1.74391750722277*AL390236.1 exp.) + (1.9361924741171*MPP7-DT exp.) + (1.56025849143997*FAM87B exp.) + (-0.578370385036099*UBE2Q1-AS1 exp.) (Fig. 2d). The heat map showed a correlation between the 19 cuproptosis-related genes and 9 lncRNA models (Fig. 2e).

**Table 2:** Clinical characteristics of bladder cancer patients in TCGA database

Feature	Type	Total %	Test %	Train %	P-value
Age	< = 65	158 (39.6)	67 (33.67)	91 (45.5)	0.0207
	> 65	241 (60.4)	132 (66.33)	109 (54.5)	
Gender	Female	102 (25.56)	49 (24.62)	53 (26.5)	0.7528
	Male	297 (74.44)	150 (75.38)	147 (73.5)	
Grade	High grade	376 (94.24)	190 (95.48)	186 (93)	0.2609
	Low grade	20 (5.01)	7 (3.52)	13 (6.5)	
	unknow	3 (0.75)	2 (1.01)	1 (0.5)	
Stage	Stage I	2 (0.5)	1 (0.5)	1 (0.5)	0.6336
	Stage II	126 (31.58)	58 (29.15)	68 (34)	
	Stage III	138 (34.59)	69 (34.67)	69 (34.5)	
	Stage IV	131 (32.83)	71 (35.68)	60 (30)	
	unknow	2 (0.5)	0 (0)	2 (1)	
T	T0	1 (0.25)	1 (0.5)	0 (0)	0.7573
	T1	3 (0.75)	1 (0.5)	2 (1)	
	T2	116 (29.07)	55 (27.64)	61 (30.5)	
	T3	190 (47.62)	97 (48.74)	93 (46.5)	
	T4	57 (14.29)	30 (15.08)	27 (13.5)	
	unknow	32 (8.02)	15 (7.54)	17 (8.5)	
M	M0	192 (48.12)	86 (43.22)	106 (53)	1
	M1	11 (2.76)	5 (2.51)	6 (3)	
	unknow	196 (49.12)	108 (54.27)	88 (44)	
N	N0	232 (58.15)	109 (54.77)	123 (61.5)	0.0956
	N1	45 (11.28)	19 (9.55)	26 (13)	
	N2	75 (18.8)	46 (23.12)	29 (14.5)	
	N3	6 (1.5)	2 (1.01)	4 (2)	
	unknow	41 (10.28)	23 (11.56)	18 (9)	

### Validation of the acquired hub CRLs by qRT-PCR

**Fig. 3:**
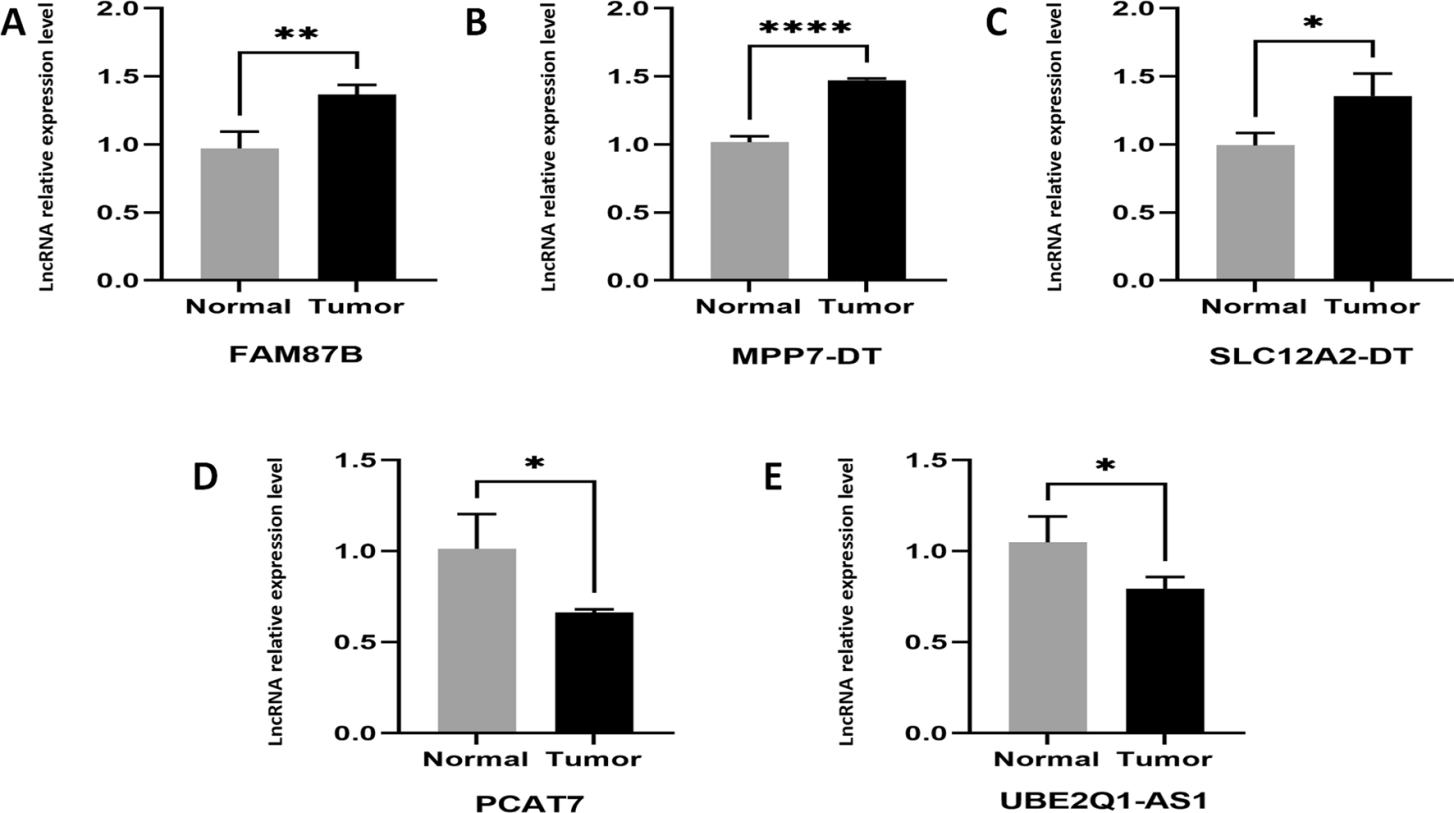
qRT-PCR validation in CRLs in human bladder cancer tissues compared to matched human bladder normal tissues (*P < 0.05, **P < 0.01, ****P < 0.001)

Five commonly used CRLs were selected for experimental verification by qRT-PCR, including PCAT7, LINC01184 (SLC12A2-DT), MPP7-DT, FAM87B and UBE2Q1-AS1 in human bladder cancer tissues and normal bladder tissues (Normal bladder tissues are assigned to the low-risk group). In addition, BX546450.2, AC021321.1, AL731537.1 and AL390236.1 primer serial numbers were not found in the literature and database, so they cannot be verified for the time being. The results showed that PCAT7 and UBE2Q1-AS1 LncRNA genes were lowly expressed in human bladder cancer tissues, while LINC01184 (SLC12A2-DT), MPP7-DT, FAM87B were expressed to a great extent in human bladder cancer tissues. Figure 3 shows the results as follows.

### The value of prognosis of CRLs signature in the train and test group

**Fig. 4:**
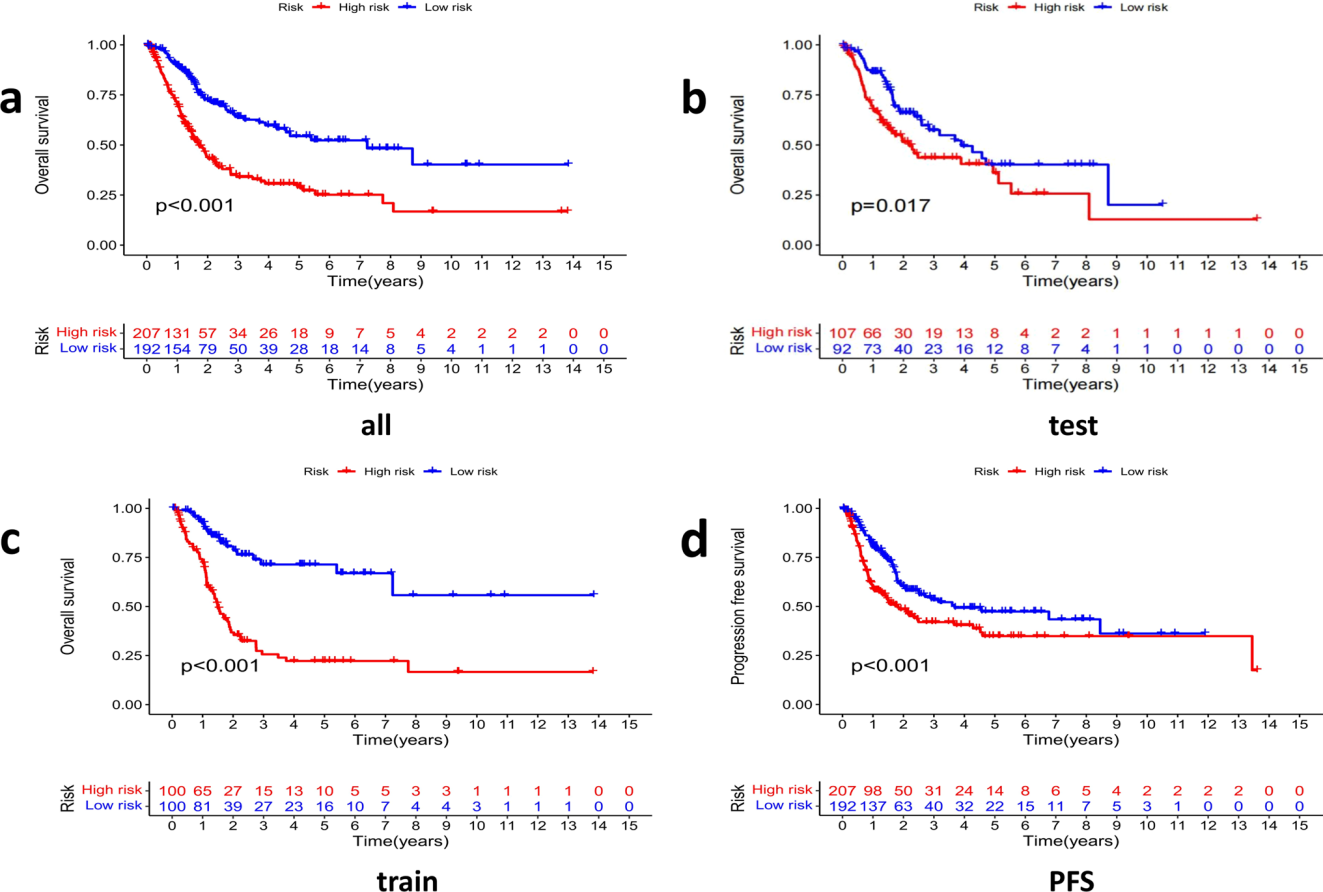
The prognostic value of lncRNA signature **a**–**d** Kaplan–Meier survival analysis compared low risk and high risk groups in all (**a**), test (**b**), train (**c**) groups, and PFS (**d**) in all samples

Risk scores for the all, train and test groups, were acquired from the above calculation formula, and all cases would be classified as low-risk group and high-risk group. Kaplan–Meier survival analysis indicated OS and progression free survival (PFS) in the low-risk group were obviously longer than those in the high-risk group (Fig. 4a–d). The result suggested that the risk formula of the 9 CRLs has a prognostic value.

**Fig. 5:**
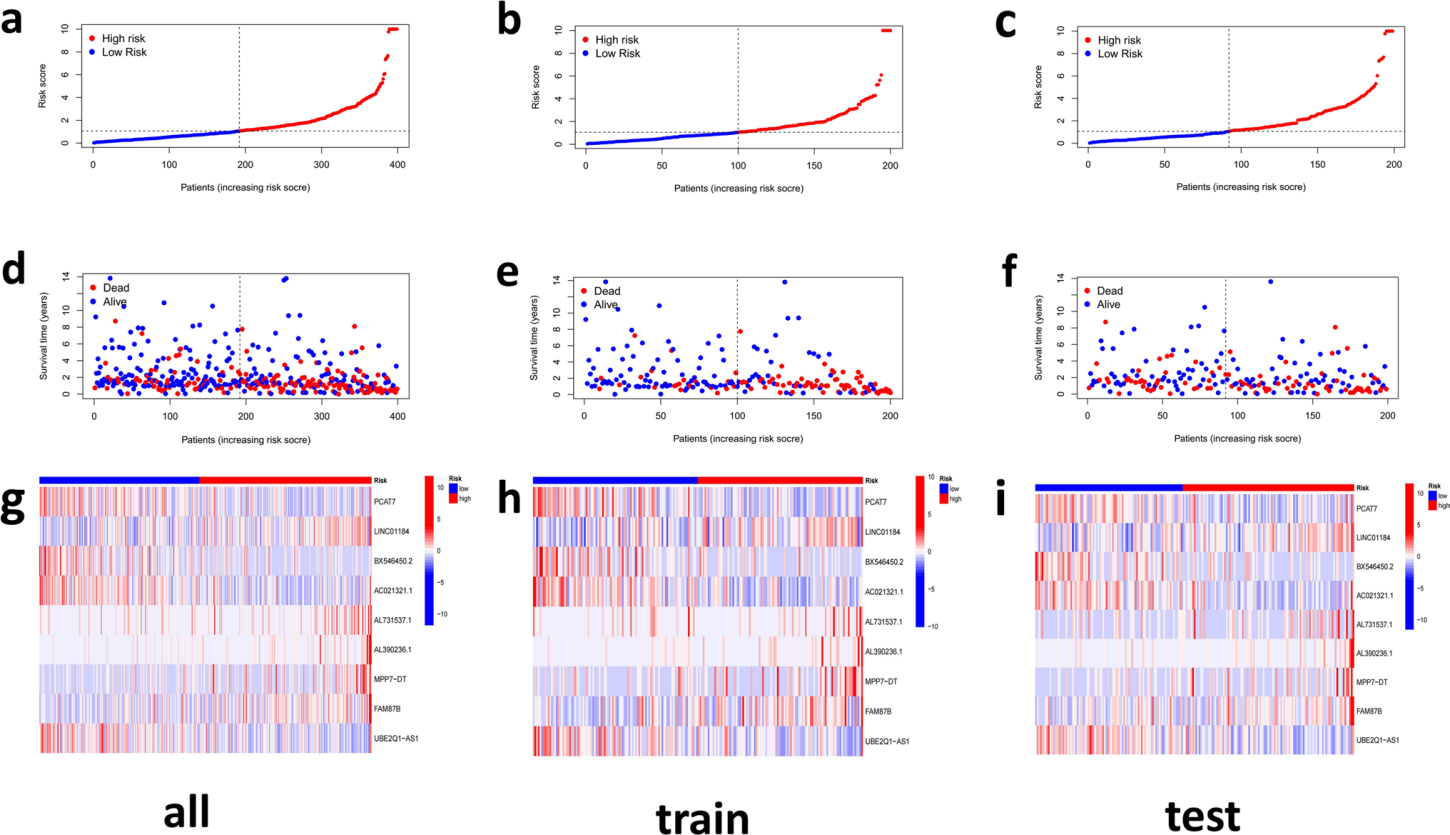
Distribution of BCa patients based on the risk score **a**–**f** Risk curve and scatter plot for the risk score and survival status of each BCa group sample. **g**–**i** Heat map showing the expression profiles of Cuprotosis-related nine-LncRNA in the all, train, test group samples

Then, Risk scores and survival status for all samples, train or test group BCa patients, are represented using prognostic curves and scatter plots (Fig. 5a–f). As the risk increased, so did the number of deaths, and a majority of the deaths were concentrated in high-risk groups (Fig. 5d–f). In addition, LINCO1184, AL731537.1, AL390236.1, AC112721.2, MPP7-DT, FAM87B were up-regulated, while PCAT7, BX546450.2, AC021321.1, UBE2Q1-AS1 were down-regulated in the high-risk group, according to the heat map of the expression profiles of candidate CRLs (Fig. 5g–i).

### Correlation between prognostic features and clinicopathological features

**Fig. 6:**
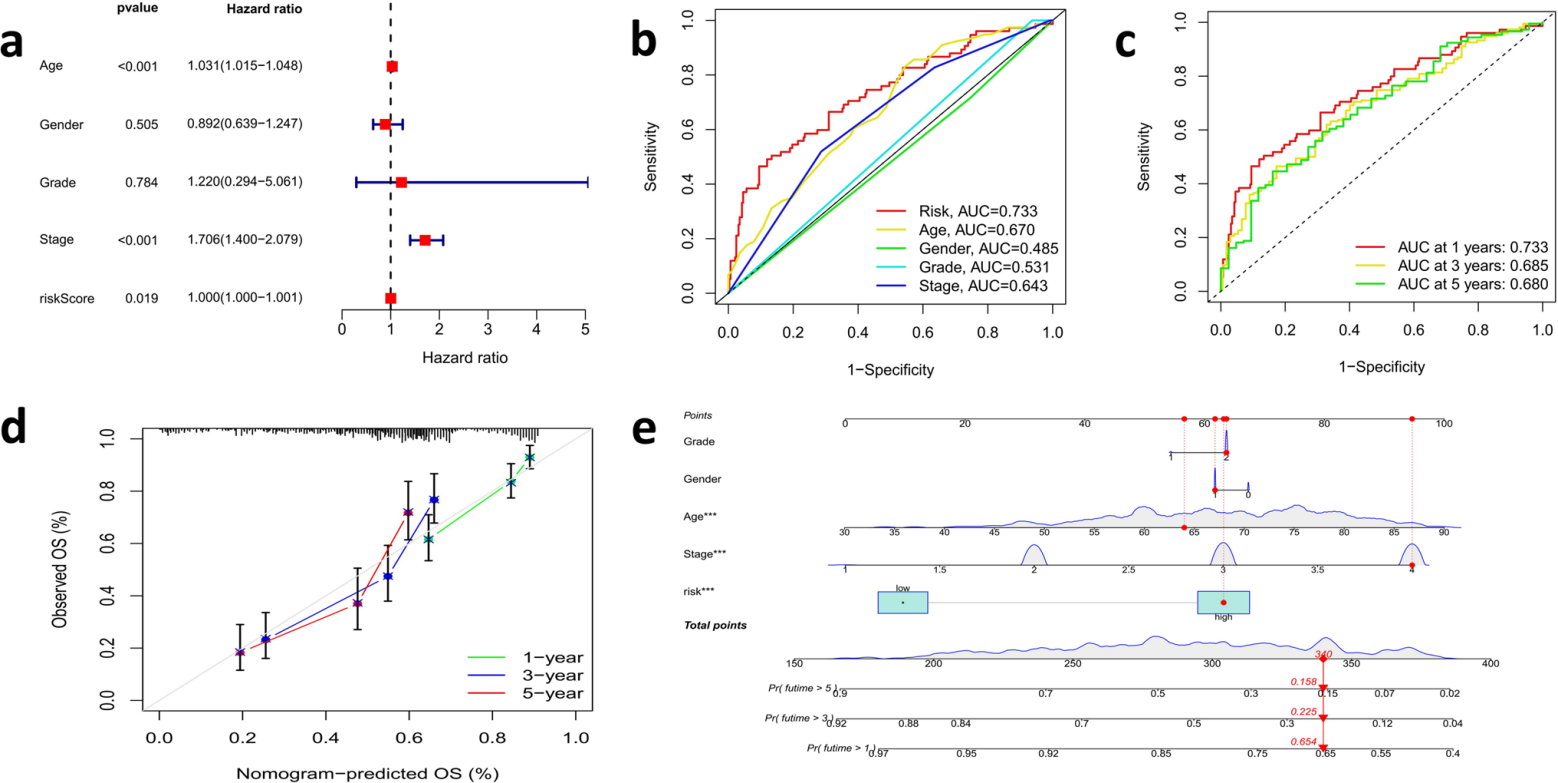
Prognostic value of CRLs signature in TCGA–BCa cohort **a** Multivariate cox regression analysis for the risk score signature as an independent prognostic factor. **b**,**c** Predictive sensitivity and specificity of risk characteristics using ROC curves. **d**, **e** A nomogram and its calibration were constructed to quantify the 1, 3, and 5 year survival probabilities by using independent predictors including tumor stage, age, gender and risk score

We used multivariate cox regression to evaluate the independence of BCa signatures. Multivariate cox regression analysis showed that the risk model could be used as an distinct prognostic factor for predicting OS in patients with BCa, which was different from other clinical characteristics (P = 0.019 < 0.05; Fig. 6a). Next, we evaluated the predictive sensitivity and specificity of the risk characteristics by using ROC curves. The area under the curve (AUC) of risk model, age, gender, grade, and stage were 0.733, 0.670, 0.485, 0.531, 0.643 (The risk model was combined with other clinical characteristics to draw ROC curves. The ROC curve threshold is 0.5, and the area greater than 0.6 indicates a more robust reliability of the results. The ROC curve area of the risk model was 0.733, the largest showed the survival prediction accuracy of the model, was the highest) (Fig. 6b). The AUC of all samples at 1, 3, and 5 years were 0.733, 0.685, and 0.680 (Fig. 6c). The areas under the ROC curve were all greater than 0.5, indicating that our predicted model was accurate in predicting the survival of patients in 1,3, and 5 years. Then, a nomogram survival prediction map and its calibration curve were built to quantify the survival probabilities of these patients by the distinct predictors, including tumor stage, age, gender and risk score (Fig. 6d, e). The survival rate was evaluated according to the risk scores of these independent predictors.

**Fig. 7:**
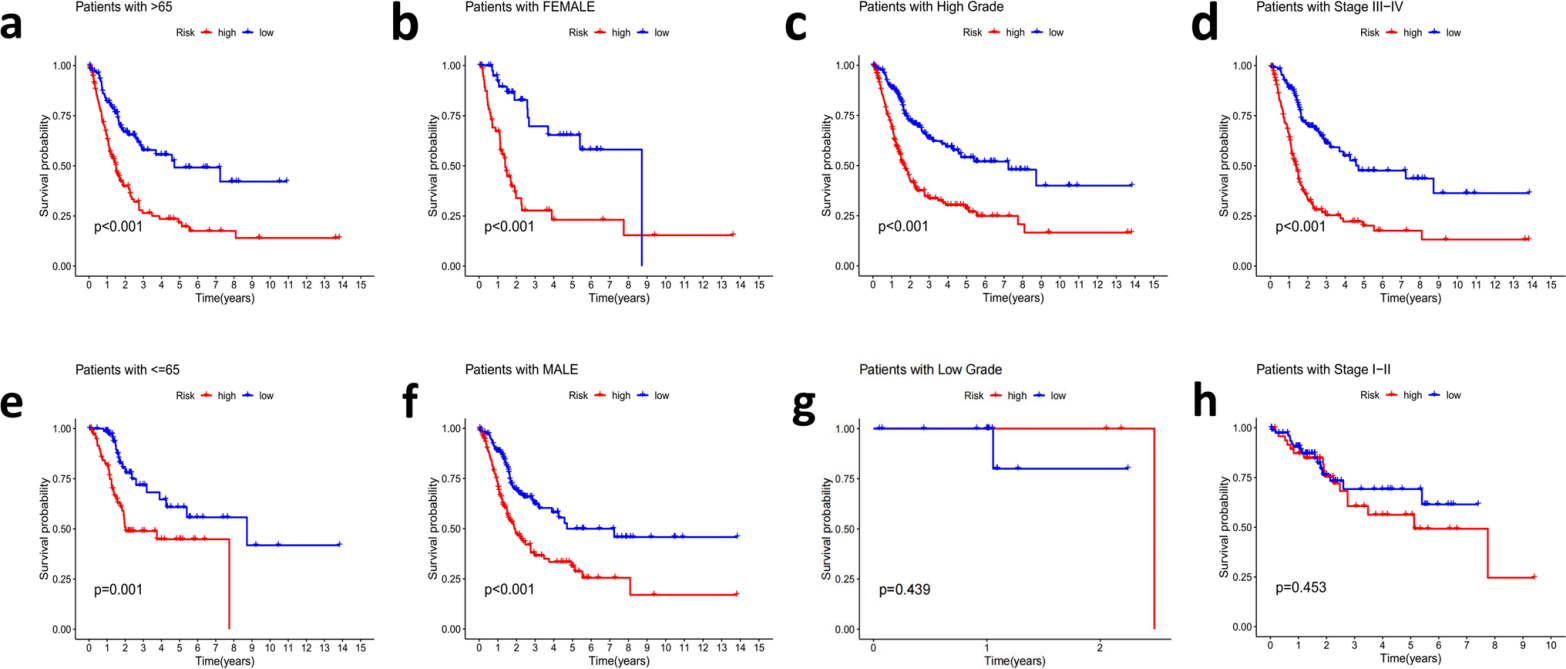
The survival prediction ability of patients with different clinical characteristics of the signature **a**–**h** Stratified analyses results of age (≤ 65 and > 65), gender (female and male), grade (1–2 and 3–4), tumor stage (I–II and III–IV)

Subsequently, Survival analyses were performed using the following clinical variables: age (≤ 65 and > 65), gender (female and male), grade (1–2 and 3–4), tumor stage (I–II and III–IV). The results showed that the OS of high-risk group was obviously below that of low-risk group. Taken together, these results confirmed the critical role of the risk scores in determining the outcome of patients with BCa (Fig. 7a–h).

**Fig. 8:**
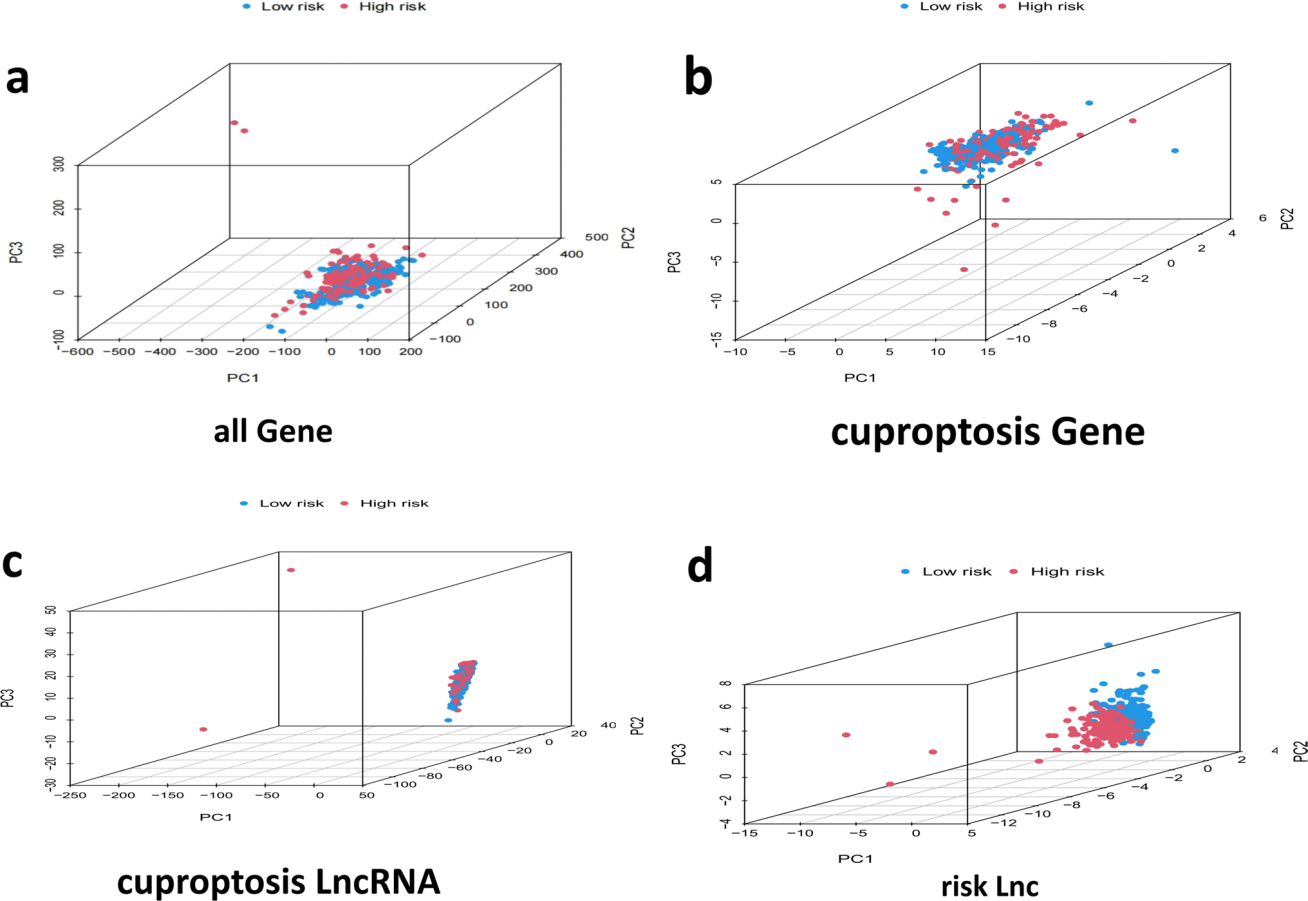
PCA analysis of different model groups in high—and low-risk patients **a** all gene group. **b** cuproptosis gene group. **c** cuproptosis lncRNA group. **d** risk lnc group. Explanation: PCA analysis results of 9-CRLs signature, which has a good degree of distinction and prediction effect between high- and low-risk groups

Finally, the result of PCA verified the classification ability of risk lncRNAs, indicating that the model had an acceptable prediction efficiency (Fig. 8a–d).

### Go and KEGG gene set enrichment analysis and immune infiltration description

**Fig. 9:**
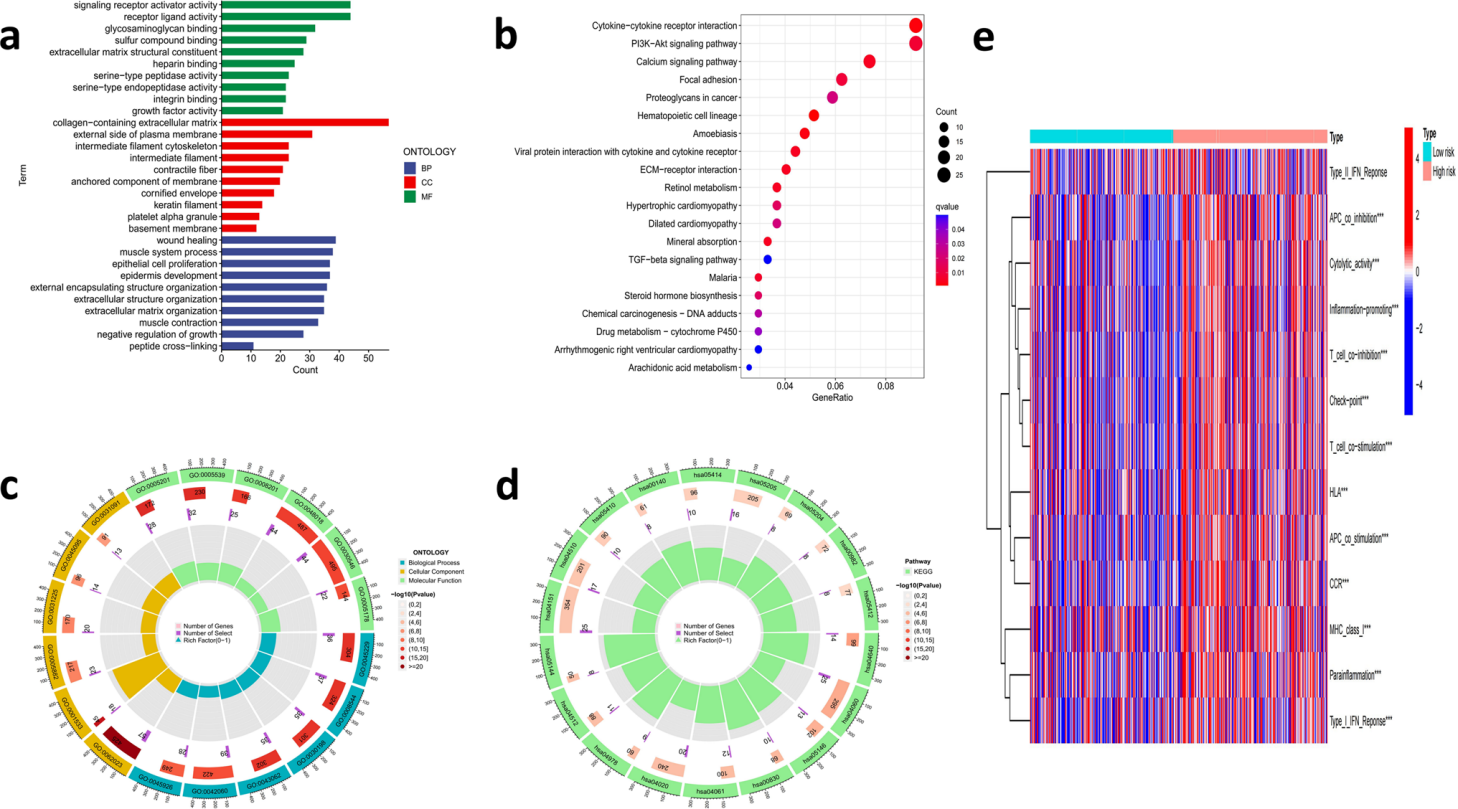
Functional enrichment analysis and immune infiltration level analysis **a**,**c** GO enrich analysis and **b**,**d** KEGG enrich analysis results. **e** Heatmap of immune infiltration based on ssGSEA analysis

According to the expression data file and risk data file, Log_2_FC = 1, FDR = 0.05 was used to obtain the differentially expressed CRLs risk genes information (Table S1). GO gene set enrichment analysis showed that 9 CRLs were significantly clustered in epithelial cell proliferation, signaling receptor activity, glycosaminoglycan binding, etc. (Fig. 9a). The KEGG gene set enrichment analysis revealed the correlated genes were enriched in cytokine-cytokine receptor interaction, PI3K-Akt signaling pathway, etc. (Fig. 9b). Then, the circle show the number of genes, number of selection and rich factors in GO or KEGG enrichment analysis (Fig. 9c, d). Subsequently, we used ssGSEA analysis to display a heat map of immune infiltration in Fig. 9e. Through the comparative analysis of immune cells and immune pathways, the differences of type I IFN response, APC co-inhibition, MHC class I, T cell co-inhibition, immune checkpoint activation, were obtained. In other words, those immune-related functions were different between high and low-risk groups, which may indirectly suggest that these genes are associated with immune responses and immune function (Fig. 9e).

### Tumor mutation burden in different risk groups based on signature and comparison of tumor immune escape

**Fig. 10:**
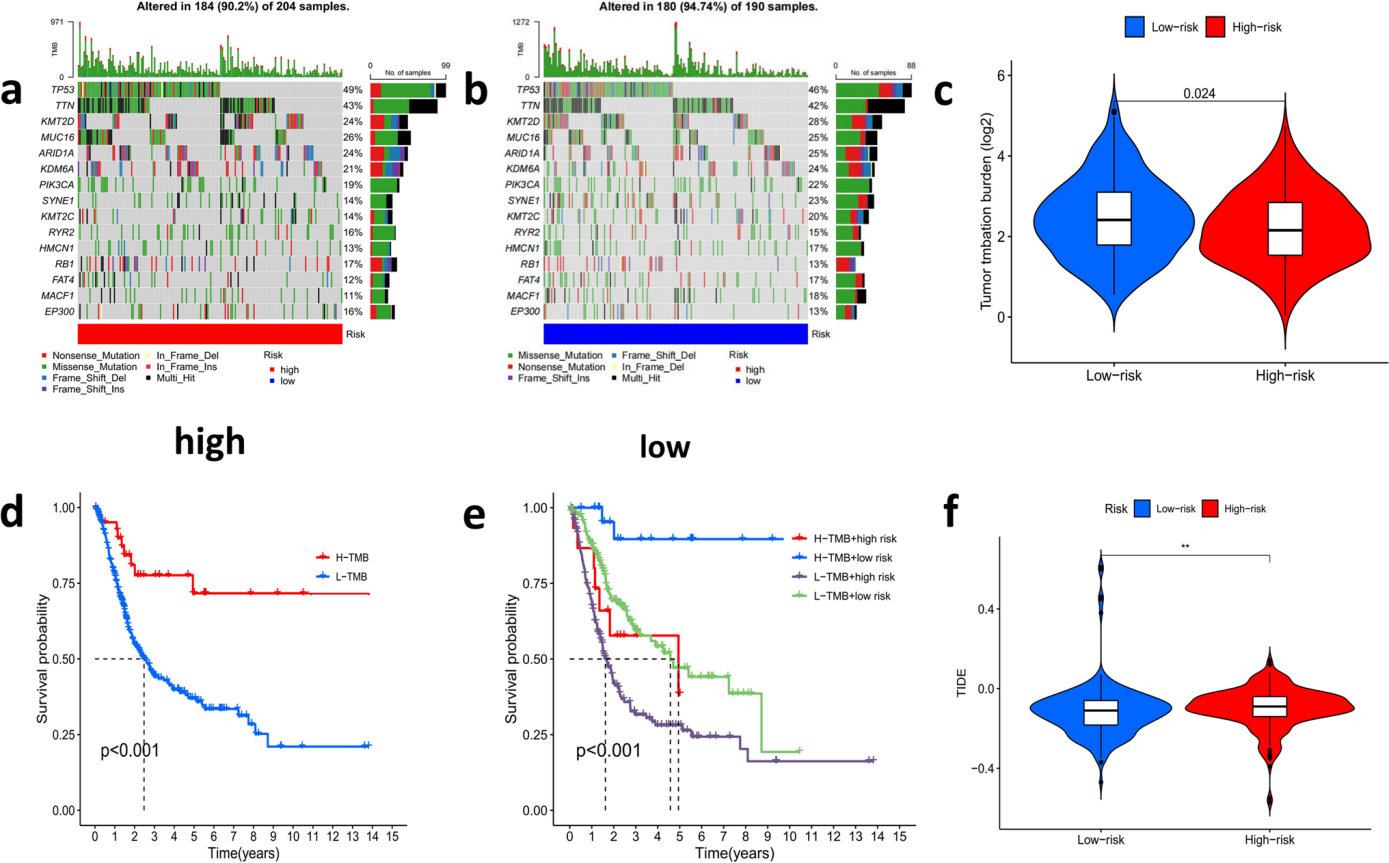
Tumor mutation burden in different risk groups based on signature and comparison of the tumor immune escape scores **a**,**b** Detailed mutation information for each gene in the high- and low-risk group. **c** The tumor mutation burden outcomes of patients in the high-low mutation group. **d** The survival outcomes of patients in the high-low mutation group. **e** The survival outcomes combination of patients in the high-low mutation load group with the high and low-risk group. **f** Tumor immune escape scores in low-risk group was significantly lower than that in high-risk group (P < 0.01)

To search the connection between risk score and TMB in BCa patients with cell mutation information. First, the waterfall diagram displayed the mutation information of each gene in detail, with different types of mutation represented by small rectangles in different colors (Fig. 10a, b). From the results, missense mutations in bladder cancer tissues were the most common, and the mutation frequency in the high-risk group was lower than that in the low-risk group. Altered in 180 (94.74%) of 190 samples of the low-risk group were higher than Altered in 184 (90.2%) of 204 samples of the high-risk group. TP53 is the gene with the highest mutation frequency. The mutation rates of TP53, TTN, KMT2D, MUC16, and ARID1A were significantly different between the two groups (Fig. 10a, b). We then used the K-M curve to compare the survival outcomes of patients in the high-low mutation group and the survival outcomes of patients in the high-low mutation load group. The results showed that the survival probability of high mutation burden group was better than that of low mutation burden group (P < 0.001) and that the worst prognosis was found in the high-risk group with the low mutation burden (P < 0.001) (Fig. 10d, e). The tumor mutation burden was lower in the high-risk group than in the low-risk group (P = 0.024 < 0.05) (Fig. 10c). In addition, tumor immune escape information was downloaded from TIDE website (http://tide.dfci.harvard.edu/), and co-analysis showed that tumor immune escape in low-risk group was significantly lower than that in the high-risk group (P < 0.01, Fig. 10f). The result indicated that there was more obvious tumor immune escape in the high-risk group, which would yield a poorer immune therapy response.

### Selection of anticancer drugs based on the sensitivity

**Fig. 11:**
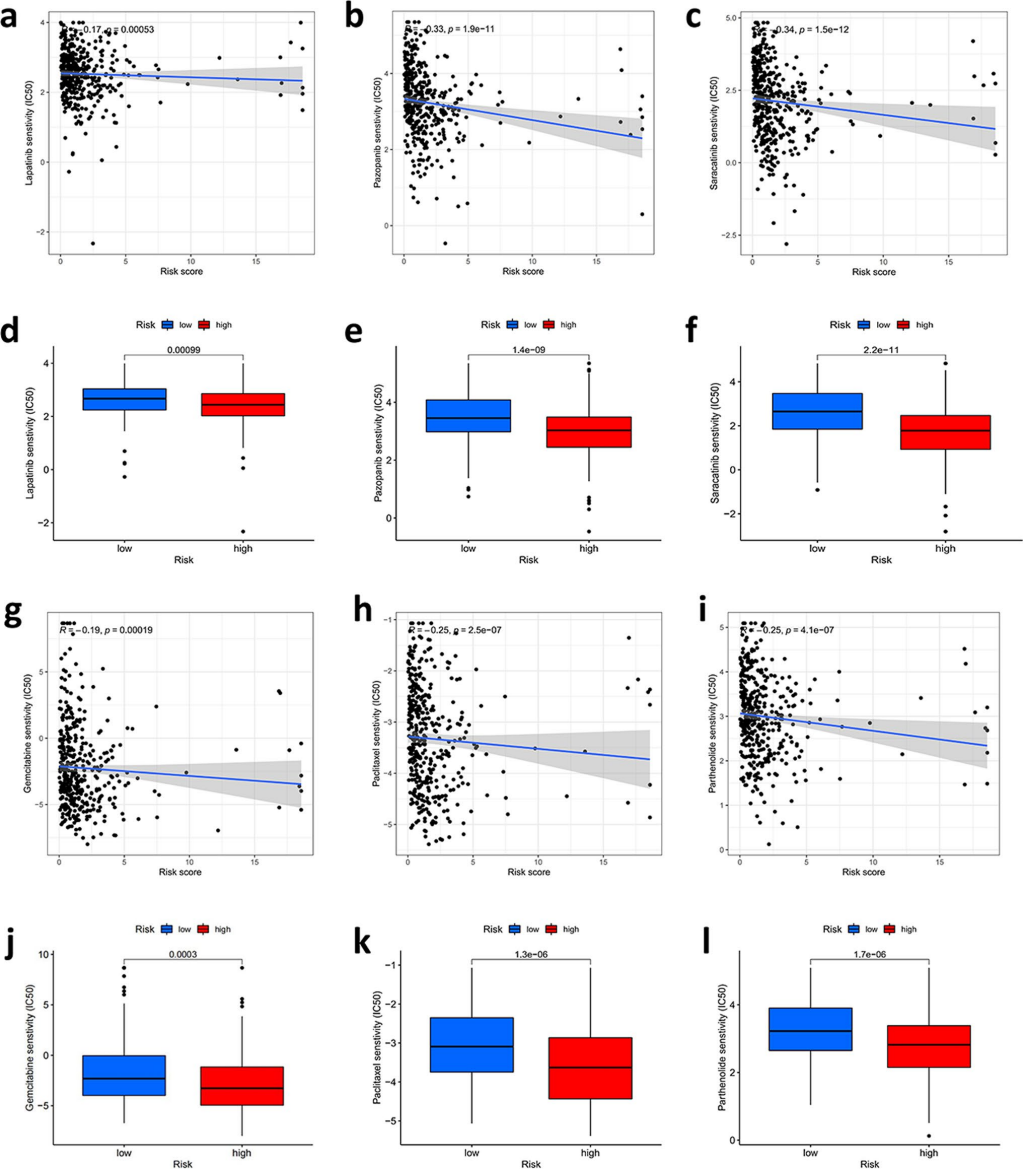
Selection of the sensitivity of patients with different risk scores to common anticancer drugs **a**–**l** The IC50 of lapatinib, pazopanib, saracatinib, gemcitabine, paclitaxel and parthenolide were all lower in the high-risk group, indicating that these drugs are more effective in the high-risk group of patients than those in the low-risk group (P < 0.001)

Then, we compared the high-risk and low-risk groups to identify potential treatment modalities for bladder cancer in terms of sensitivity to anticancer drugs. The results showed that the IC50 of several antitumor medications approved by FDA registration, including lapatinib, saracatinib, gemcitabine, paclitaxel and palenolactone, were all lower in the high-risk group, suggesting these drugs are more functional in the high-risk group of patients (P < 0.001, Fig. 11a–l). And this further explains that these drugs can potentially be used to treat bladder cancer patients.

## Discussion

Currently, some advances have been made progress in the treatment of BCa patients, but the prognosis is still unsatisfactory with patients of advanced and metastatic BCa. Some researchers had studied molecular markers related to tumor prognosis and drug susceptibility and found that although some patients had similar TNM stages or risk factors [28–30]. Moreover, enormous studies have corroborated that lncRNA participates in multifarious critical physiological processes and is intimately relevant to the occurrence and development of tumors. Applying lncRNAs as biomarkers or intervention targets could provide new insights into predicting prognosis, survival and treatment outcome [31, 32]. Many studies have shown that lncRNAs play an important role in BCa, and these experimental researches suggested that lncRNAs were associated with the proliferation and invasion of bladder cancer, indicating that they may serve as potential biomarkers for targeted therapy in bladder cancer [20–24].

Cuproptosis is the latest form of death to be discovered independently of other forms. FDX1 and the abundance of acylated proteins, key elements for cuproptosis, are highly related to many human tumors. According to the latest studies, copper induces cell death mainly by binding directly to the acylated components of the tricarboxylic acid cycle in vivo and subsequently causing toxic protein stress [11]. Cu is involved in a variety of biological processes. A number of studies have shown that the Cu ion level in tumor patients, both in serum and tissue, is significantly higher than that in healthy people recently [33, 34]. Therefore, cupric ion carriers may be a potential therapeutic approach to kill cancer cells with this metabolic property, which indirectly explains the association with tumor proliferation, invasion, and metastasis, and that includes bladder cancer. The results of clear cell renal cell carcinomas (ccRCC) may be greatly influenced by these cuproptosis-related genes, according to Zilong Bian. For the prediction of OS and PFS in ccRCC patients, the predictive risk score based on the expression signature of CRGs performed well and was substantially correlated with immune infiltration levels and PD1 expression [35]. In hepatocellular carcinoma, FDX1 was considerably downregulated, and high expression was linked to a prolonged survival time [36]. Lung cancer risk was shown to be elevated by the minor alleles of SLC31A1-rs10981694 and FDX1-rs10488764. In contrast, a lower incidence of the condition was associated with the minor alleles of rs9535826 and rs9535828 in ATP7B [37]. By using and analyzing a public database, some researchers investigated the relationship between cuproptosis-related genes and the prognosis of melanoma. They discovered that 11 out of 12 genes were upregulated in melanoma tissues and that three genes (LIPT1, PDHA1, and SLC31A1) have predictive value for the prognosis. Longer overall survival was seen in the subgroup of melanoma patients with higher cuproptosis-related gene expression compared to those with lower gene expression [38]. Since both cuproptosis and lncRNA were gone through in tumor regulation, the regulatory mechanism of upstream and downstream genes and their relationship with tumor occurrence and development could be better studied by using cuproptosis genes and their co-expression of lncRNAs. Thus, a better understanding and identification of cuproptosis-related lncRNA predictive signals would be of great significance for BCa patients.

In our study, we identified nine CRLs (PCAT7, LINC01184(SLC12A2-DT), BX546450.2, AL731537.1, AL390236.1, AC021321.1, MPP7-DT, FAM87B, UBE2Q1-AS1) for inclusion in a predictive signature. Subsequently, we applied this model to evaluate each patient's risk scores and divided BCa patients into low-risk group and high-risk group according to the median risk score. The overall survival time of the low-risk group was significantly higher than that of the high-risk group, and the ROC curve drawn subsequently also showed that the model had good predictive function, which was also confirmed in our collected samples. We then focused on the up-regulated lncRNA genes in the prediction model, including LINC01184, AL731537.1, AL390236.1, MPP7-DT, FAM87B. Among them, studies had shown that LINC01184 was significantly upregulated in colon cancer tissues and cells, and its up-regulation was positively related to CRC progression [39], suggesting that it may be a high-risk factor or a negative prognostic factor in cancer. The remaining four lncRNAs were less studied, and we will continue to pay attention to them. The cuproptosis-related risk profiles for lncRNA may aid in determining the prognosis and molecular profile of patients with ccRCC, lung adenocarcinoma, and hepatocellular carcinoma as well as improving therapy choices that may be used in the clinic [40–42]. According to the current study, a novel signature based on the cuproptosis gene may be able to predict a patient's prognosis, biological makeup, and the best course of therapy for them if they have glioma or triple negative breast cancer [43, 44]. The immune microenvironment and tumor immunotherapy may both be affected by cuproptosis-related lncRNAs at the same time [45–47].

In addition, we calculated TMB for the high- and low-risk groups, and the result showed that the main mutant genes were TP53, TTN, KMT2D and MUC16. Among them, TP53 mutation frequency was found to be the highest in the high-risk group, and higher than that in low-risk group suggesting that it may be a negative prognostic regulator, as mentioned in some of the literature [48]. The above results indirectly demonstrate the reliability of our risk model in predicting prognosis. Moreover, researchers found that TP53, TTN and KMT2D had the highest mutation rates in bladder cancer, and TP53 could forecast the OS rate and treatment response of muscle-invasive BCa [49, 50], suggesting that TP53 gene might become a target of new drugs for BCa patients in the future. Wang reported that MUC16 and TTN showed high mutation frequency in the groups with high cuproptosis score of the patients with gliomain [43]. In addition, Li found that patients with oral squamous cell carcinomain high-risk group were prone to TTN mutations [51].

Moreover, our study found that cuproptosis-related lncRNAs might be associated with immunizing response or function. GO and KEGG gene set enrichment analysis of all differentially expressed genes (Table S1) in the two risk groups indicated that these differentially expressed genes (DEGs) were chiefly involved in epitheial cell proliferation, calcium signaling pathway, cytokine-cytokine receptor interaciton, and PI3K-AKT signaling pathway. These functions and pathways played essential roles in the recurrence, progression, and metastasis of BCa and might be associated with the intensity of cuproptosis or cuproptosis related to counter-cyclical regulation [52–55]. Among them, PI3K had been proven to be a classical immune regulatory pathway, and the effective target regulated the immune response through the PI3K-AKT signaling pathway [56]. And it had been reported that immune infiltration would increase if PI3K pathway was inhibited [57, 58]. These results indicated that the PI3K-AKT signaling pathway enriched and screened by CRLs was a meaningful pathway in immune regulation, which indirectly indicated that CRLs were related to immune response.

In addition, from the current study, we knew that cancer cells tend to prevent the attack of the immunity system, and activate the immune checkpoint to suppress the immune system attacks, thereby inhibiting the function of immune cells such as CD4, CD8, and APC. The use of immune checkpoint inhibitors could inhibit the activation and restore immune regulating function, and that immune checkpoint inhibitor was used to treat bladder cancer has been proven to be effective [59, 60]. Immune function analysis of CRLs suggested the high-risk group of APC cells and T cells functions inhibitory increases, and increased activation of the immune checkpoints. The results suggested that the immune function of high-risk group patients was suppressed and the anti-tumor immunity was decreased, which indicated that the CRLs we screened were related to the immune response. The application of checkpoint inhibitors or corresponding immunotherapy may benefit the prognosis of patients with bladder cancer in the coming years.

Finally, we performed a comparative analysis of anticancer drug sensitivity between the low-risk and high-risk groups to help identify potential therapeutic modalities or drug options for BCa patients. Lapatinib, pazopanib, saracatinib, gemcitabine, paclitaxel and palenolactone were more sensitive to patients in the high-risk group. Most of these drugs were targeted drugs, and some of these drugs had been reported to have immune-boosting effects in other advanced tumors [61–63] and may be beneficial in advanced bladder cancer as well.

Our study showed an advantage in the systematic analysis obtained from TCGA cohorts and the evaluation of CRLs in BCa. Subsequently, we performed a small samples of human bladder cancer tumor tissues validation, and the results were in line with our expectations. Of course, our research has some limitations at present. For example, the number of samples needs to be increased, the methods need to be more diversified, and the experiments on cells, animals and humans need to be carried out successively. With in-depth research, we believe that our prediction model will be more perfect and reliable.

## Conclusion

In a word, we identified a CRLs signature, constructed by a integrative analysis of bioinformatics, that was significantly related to the prognosis in patients of BCa. Furthermore, this risk score is an independent risk factor for the prognosis of BCa and can guide the selection of targeted or immune-boosting therapy drugs in clinical practice.

## Supplementary Information

Additional file1 (DOCX 145 KB)

Additional file2 (DOCX 11 KB)

## Data Availability

The original contributions appearing in the study report are included in the article or supplementary materials, and the data are from the TCGA database (https:// portal. gdc. cancer.gov), TIDE (http://tide.dfci.harvard. edu/). Our data and materials will be used for non-commercial purposes and will be freely available to scientists. For further enquiries, please contact the corresponding author.
