# Supplementary material for: Identification and validation of a novel cuproptosis-related lncRNA gene signature to predict prognosis and immune response in bladder cancer
Source: Discov Oncol. 2022 Dec 1;13:133. doi: 10.1007/s12672-022-00596-w (PMC9715909; doi:10.1007/s12672-022-00596-w)
Supplement: Supplementary file 1 — Additional file1 (DOCX 145 KB) [file 12672_2022_596_MOESM1_ESM.docx]

| **Table S1 The differentially expressed CRLs risk genes information** |
| --- |

| gene | lowMean | highMean | logFC | pValue | fdr |
| --- | --- | --- | --- | --- | --- |
| TMEM178A | 4.110455208 | 1.808925121 | -1.184165483 | 7.84E-10 | 1.46E-08 |
| RAB26 | 6.048321875 | 2.858605475 | -1.081223395 | 0.022442238 | 0.039149808 |
| KRT5 | 916.0571854 | 2754.016389 | 1.588027578 | 1.22E-06 | 7.81E-06 |
| SRCIN1 | 7.913073958 | 3.766171498 | -1.071139545 | 2.90E-15 | 4.68E-13 |
| MROH3P | 2.748124479 | 1.050091787 | -1.387931916 | 1.60E-08 | 1.86E-07 |
| AC104530.1 | 0.803282813 | 1.62683124 | 1.018084687 | 0.000872063 | 0.002322513 |
| LINC02593 | 2.945815625 | 1.469564573 | -1.003278384 | 0.000276417 | 0.000860731 |
| MSRB3 | 7.022815104 | 14.43276473 | 1.039226331 | 1.22E-09 | 2.13E-08 |
| LINC00165 | 1.315110938 | 2.670469726 | 1.021909025 | 0.025328688 | 0.043351176 |
| KANK4 | 1.881408333 | 3.829199839 | 1.025229954 | 1.15E-10 | 2.88E-09 |
| OSMR | 18.05807188 | 37.17685475 | 1.041760861 | 1.49E-15 | 2.67E-13 |
| ID4 | 41.69783229 | 20.76377053 | -1.005903936 | 0.010949378 | 0.021101145 |
| AL390294.1 | 1.665749479 | 0.573667472 | -1.537884821 | 1.07E-12 | 5.64E-11 |
| S100A10 | 787.5970828 | 1582.205078 | 1.006406935 | 9.49E-13 | 5.11E-11 |
| TGFBI | 95.53416302 | 204.0945076 | 1.09514872 | 5.61E-11 | 1.55E-09 |
| CCL24 | 2.661359896 | 6.618679388 | 1.314379768 | 6.27E-05 | 0.000237738 |
| CACNA1D | 2.207330208 | 0.929720129 | -1.247434071 | 1.05E-11 | 3.79E-10 |
| AP000346.2 | 1.566171875 | 0.746316103 | -1.069383825 | 8.86E-16 | 1.66E-13 |
| NCF1C | 2.559760417 | 5.278302415 | 1.044065225 | 0.000151063 | 0.000511684 |
| IL11 | 4.495643229 | 13.14099324 | 1.547474869 | 0.001058322 | 0.002745548 |
| MYL9 | 236.6985339 | 482.1517947 | 1.026436648 | 4.95E-07 | 3.60E-06 |
| EMP1 | 36.08833646 | 87.05781643 | 1.270441193 | 1.68E-14 | 2.05E-12 |
| CLDN6 | 1.592565104 | 11.74759807 | 2.882941555 | 0.000163748 | 0.000548167 |
| CPA3 | 8.415219271 | 19.69395749 | 1.22668028 | 6.86E-07 | 4.76E-06 |
| GPAT3 | 1.827416667 | 4.89786248 | 1.422346649 | 3.03E-14 | 3.18E-12 |
| TAGLN | 84.53806719 | 181.5301219 | 1.102535926 | 1.22E-07 | 1.07E-06 |
| ARHGAP24 | 1.096389063 | 2.327554106 | 1.086054864 | 7.85E-13 | 4.40E-11 |
| SPRR2A | 41.53855417 | 173.4354461 | 2.061875875 | 0.000813967 | 0.002193197 |
| NIBAN1 | 13.24837708 | 26.71390386 | 1.01177518 | 3.69E-13 | 2.37E-11 |
| IL1R2 | 3.695159896 | 10.09583977 | 1.450052221 | 3.82E-06 | 2.09E-05 |
| GPC2 | 8.732079167 | 2.978887601 | -1.551551521 | 2.54E-13 | 1.74E-11 |
| CECR2 | 3.248591667 | 1.618738325 | -1.004944627 | 4.73E-07 | 3.46E-06 |
| NUAK2 | 7.409528646 | 15.24869887 | 1.041232473 | 5.52E-07 | 3.95E-06 |
| CYP4Z1 | 1.415956771 | 0.658162802 | -1.105260825 | 2.16E-10 | 4.86E-09 |
| KLK5 | 15.82817292 | 66.91270274 | 2.079785387 | 3.98E-08 | 4.05E-07 |
| IDSP1 | 1.707767188 | 0.671545411 | -1.346554447 | 4.74E-11 | 1.34E-09 |
| CYP1A2 | 40.15619792 | 4.556214171 | -3.139715209 | 0.001355304 | 0.003422437 |
| RFLNA | 2.553728646 | 5.506996296 | 1.108660403 | 1.07E-05 | 5.09E-05 |
| S100A3 | 15.61083385 | 175.7212116 | 3.492668846 | 0.004985112 | 0.01066055 |
| ELOVL4 | 2.916270833 | 6.68377037 | 1.19653746 | 2.16E-08 | 2.41E-07 |
| KCNH4 | 1.698814063 | 0.773182448 | -1.135647164 | 1.35E-06 | 8.49E-06 |
| NCR3 | 0.711443229 | 1.489119646 | 1.06563913 | 9.85E-05 | 0.000352645 |
| CIDEC | 0.516177604 | 2.586364251 | 2.324986019 | 3.26E-09 | 4.88E-08 |
| SNORD123 | 18.66268542 | 7.711016747 | -1.275163587 | 2.90E-17 | 1.25E-14 |
| TNXB | 1.168464583 | 3.314662963 | 1.504248178 | 0.000108397 | 0.000383228 |
| PPP1R14C | 15.3263474 | 30.90115684 | 1.011646937 | 0.000652548 | 0.001802906 |
| ACP5 | 41.23177292 | 95.0450686 | 1.204855278 | 7.06E-07 | 4.88E-06 |
| C5orf66-AS1 | 1.258547917 | 3.224831401 | 1.357463591 | 0.001001505 | 0.002618125 |
| FOXI1 | 13.79666406 | 0.361755717 | -5.25315985 | 0.00099091 | 0.002592502 |
| OTOF | 2.059964583 | 0.837230435 | -1.298922871 | 5.78E-10 | 1.12E-08 |
| RHOH | 1.447836458 | 3.160793559 | 1.126388161 | 1.16E-06 | 7.48E-06 |
| SLURP1 | 26.69279479 | 4.560008535 | -2.549341937 | 0.005462686 | 0.011542709 |
| CCN3 | 3.748233854 | 8.198473269 | 1.129144308 | 0.00035647 | 0.001068038 |
| PCAT7 | 4.181267188 | 1.716112238 | -1.284796324 | 2.08E-16 | 5.13E-14 |
| AC034213.1 | 0.640020313 | 1.774899034 | 1.47154736 | 0.002715619 | 0.006281287 |
| ANKRD1 | 1.695875 | 3.437629147 | 1.019384079 | 0.000280519 | 0.000871843 |
| ATP1A4 | 7.606569271 | 3.210861192 | -1.244285616 | 1.43E-10 | 3.48E-09 |
| B4GALNT4 | 20.96273281 | 9.662264251 | -1.117393592 | 6.09E-05 | 0.000231887 |
| ISL1 | 0.580301563 | 2.258152657 | 1.960268301 | 0.001858795 | 0.004513703 |
| GALNT17 | 1.029292708 | 2.836084058 | 1.462246981 | 1.06E-07 | 9.49E-07 |
| FHL1 | 15.0364349 | 33.11611111 | 1.139070716 | 8.83E-07 | 5.88E-06 |
| NPIPB15 | 12.6151349 | 6.220778905 | -1.019988496 | 2.24E-06 | 1.31E-05 |
| CARD17 | 0.645744271 | 1.569341546 | 1.281124526 | 0.00013534 | 0.000464695 |
| PTGIS | 11.49017031 | 29.51717407 | 1.361154424 | 8.69E-06 | 4.25E-05 |
| LAMA3 | 20.92604115 | 42.32561304 | 1.01623156 | 2.06E-07 | 1.68E-06 |
| NCF1B | 1.086523958 | 2.213152818 | 1.026383086 | 0.000802672 | 0.002164908 |
| LMOD1 | 18.07903125 | 40.68193398 | 1.170070891 | 0.000202829 | 0.000660967 |
| GPR1 | 1.563733854 | 3.198232206 | 1.0322797 | 8.68E-08 | 8.02E-07 |
| PPM1N | 13.94196875 | 5.082739614 | -1.45575607 | 1.09E-08 | 1.35E-07 |
| C6orf15 | 2.748365104 | 21.00680676 | 2.9342113 | 0.000393264 | 0.001161855 |
| RNU6-403P | 2.626309375 | 5.874332045 | 1.161387943 | 0.002594112 | 0.006022428 |
| AP001205.1 | 1.427609375 | 0.64360161 | -1.14936144 | 3.90E-11 | 1.14E-09 |
| AL161431.1 | 6.302439583 | 21.72986586 | 1.785696981 | 8.87E-08 | 8.18E-07 |
| ZNF350-AS1 | 17.88707656 | 8.76553913 | -1.029002882 | 0.000131731 | 0.000454597 |
| PITX1 | 32.07383906 | 69.92186699 | 1.12434666 | 1.95E-08 | 2.22E-07 |
| LINC02672 | 37.03092969 | 13.37796812 | -1.468871758 | 3.18E-09 | 4.79E-08 |
| AC138904.1 | 1.928205729 | 0.859270853 | -1.166074123 | 6.00E-06 | 3.11E-05 |
| LINC00967 | 13.4520625 | 5.890133494 | -1.19145515 | 1.21E-09 | 2.13E-08 |
| TUBAL3 | 0.642766667 | 1.448604831 | 1.172297073 | 0.000335588 | 0.001017045 |
| HTR3A | 0.317907292 | 2.70080934 | 3.086713786 | 1.88E-07 | 1.56E-06 |
| KRT24 | 1.174952604 | 40.33489195 | 5.101353924 | 0.012946801 | 0.024341238 |
| LGI2 | 1.307243229 | 3.618735105 | 1.468957907 | 1.20E-05 | 5.61E-05 |
| CDO1 | 1.082142188 | 3.899571337 | 1.84942547 | 3.76E-06 | 2.06E-05 |
| TRH | 0.042552083 | 9.935118196 | 7.867163553 | 0.022372117 | 0.039048339 |
| CEMIP | 5.132229688 | 14.60785829 | 1.509087032 | 0.000280222 | 0.000871084 |
| HMGA2 | 2.407302604 | 6.688955072 | 1.474363351 | 6.21E-10 | 1.19E-08 |
| RAET1L | 1.633127083 | 6.351389372 | 1.959435157 | 0.000146839 | 0.000499213 |
| CYP4F23P | 28.32325833 | 9.426255072 | -1.587230618 | 8.11E-18 | 5.29E-15 |
| FAM227A | 1.470250521 | 0.724908374 | -1.020191442 | 7.77E-12 | 2.95E-10 |
| NOG | 1.373663542 | 3.918600161 | 1.512309693 | 0.024885069 | 0.042703846 |
| WNT11 | 4.565465625 | 9.408578905 | 1.043214825 | 2.07E-06 | 1.22E-05 |
| TESC | 56.6342375 | 27.19586232 | -1.058287308 | 9.99E-07 | 6.55E-06 |
| KRT79 | 4.142902083 | 13.68533092 | 1.723916692 | 0.022261149 | 0.03888792 |
| LRRC38 | 0.327161458 | 3.783172947 | 3.531522025 | 0.001963014 | 0.004734379 |
| NNAT | 156.8665448 | 34.85020435 | -2.170298678 | 0.021047846 | 0.037057922 |
| LMNTD2-AS1 | 1.648963542 | 0.769350564 | -1.099846467 | 8.63E-11 | 2.24E-09 |
| IGKJ3 | 3.1831875 | 7.965944122 | 1.323373221 | 0.004957888 | 0.010610119 |
| HEPACAM2 | 7.466845833 | 0.556590016 | -3.745812009 | 0.004665551 | 0.010064198 |
| TLX1 | 2.490747396 | 0.976960548 | -1.350206507 | 0.000105972 | 0.000375877 |
| FKBP9P1 | 1.299929688 | 2.846253784 | 1.130630714 | 7.07E-11 | 1.88E-09 |
| CGB5 | 1.243607292 | 22.54212432 | 4.180020592 | 0.017583727 | 0.031725344 |
| PPP2R2C | 2.109520313 | 4.573094847 | 1.116255862 | 0.003235814 | 0.007311548 |
| OMD | 1.118151042 | 2.349805636 | 1.071426346 | 6.19E-06 | 3.19E-05 |
| AC091544.4 | 1.763596354 | 0.619049275 | -1.510394244 | 6.88E-17 | 2.25E-14 |
| PTX3 | 4.948265104 | 15.06331562 | 1.60604466 | 4.06E-05 | 0.000163296 |
| LRFN2 | 2.235313021 | 0.966931884 | -1.208990705 | 7.05E-13 | 4.04E-11 |
| AOC2 | 7.167068229 | 3.420130757 | -1.067331606 | 1.58E-15 | 2.79E-13 |
| DES | 206.2821578 | 680.3450003 | 1.721647475 | 1.70E-06 | 1.03E-05 |
| AC004148.1 | 7.117421354 | 3.544389855 | -1.005817349 | 1.72E-19 | 3.45E-16 |
| AP003068.4 | 2.881228646 | 1.402355395 | -1.03883214 | 5.07E-09 | 7.01E-08 |
| KRT14 | 621.442625 | 2287.941974 | 1.880357359 | 7.19E-05 | 0.000267512 |
| LINC01768 | 2.156108333 | 0.393554911 | -2.453792818 | 7.18E-06 | 3.62E-05 |
| FER1L4 | 116.5615484 | 35.70325491 | -1.706964438 | 6.07E-21 | 3.30E-17 |
| AC023421.2 | 4.92043125 | 1.876251852 | -1.39093127 | 6.01E-11 | 1.63E-09 |
| PLA2G2F | 73.78626979 | 28.70280081 | -1.362160862 | 4.08E-13 | 2.55E-11 |
| BARX2 | 6.048092708 | 19.66047746 | 1.700746199 | 5.90E-05 | 0.000225327 |
| HPGD | 220.2254755 | 107.080339 | -1.040287757 | 3.78E-06 | 2.07E-05 |
| MT1A | 3.915984896 | 19.23552625 | 2.296326194 | 1.03E-06 | 6.72E-06 |
| NOD2 | 1.505197917 | 3.120282287 | 1.051723355 | 1.34E-06 | 8.42E-06 |
| DNASE1L3 | 0.988420833 | 5.3844219 | 2.445594133 | 2.74E-10 | 5.95E-09 |
| SERPINB4 | 30.82845208 | 73.6028087 | 1.255498368 | 9.56E-06 | 4.61E-05 |
| MALL | 5.571095833 | 12.95417923 | 1.217384571 | 2.57E-10 | 5.64E-09 |
| GPR68 | 9.307594792 | 19.98245185 | 1.102253304 | 2.94E-11 | 8.99E-10 |
| IGHV1-69-2 | 6.557986979 | 27.61850177 | 2.074310117 | 0.027205079 | 0.046046091 |
| AC010636.1 | 3.602620833 | 1.027416103 | -1.810026229 | 0.00012958 | 0.000448216 |
| NRG1 | 0.794840625 | 2.18489066 | 1.458823567 | 7.22E-07 | 4.97E-06 |
| PTPRR | 16.04427604 | 7.530033816 | -1.091330444 | 1.05E-07 | 9.46E-07 |
| AC004597.1 | 1.577719792 | 0.519075523 | -1.603824635 | 1.61E-08 | 1.88E-07 |
| RNF186 | 2.699371354 | 0.974071659 | -1.470523648 | 5.50E-08 | 5.37E-07 |
| RANP4 | 1.460539063 | 0.720754911 | -1.018920276 | 1.32E-10 | 3.25E-09 |
| TENM2 | 2.95004375 | 7.311030274 | 1.309338376 | 6.84E-11 | 1.83E-09 |
| XPNPEP2 | 0.581835938 | 1.682980515 | 1.532334161 | 7.19E-08 | 6.78E-07 |
| GDA | 3.997455208 | 1.554969726 | -1.362195376 | 0.004221952 | 0.009213226 |
| WDFY4 | 0.895843229 | 2.062422866 | 1.203021974 | 9.71E-07 | 6.39E-06 |
| DUSP15 | 2.147234375 | 1.030844928 | -1.058652352 | 0.000507098 | 0.001442803 |
| GAS6 | 28.61726875 | 63.79183881 | 1.156485878 | 1.97E-08 | 2.24E-07 |
| LINC01833 | 5.690783854 | 2.4597657 | -1.210106483 | 5.03E-07 | 3.64E-06 |
| YJEFN3 | 7.141802604 | 3.494389211 | -1.031247952 | 1.95E-11 | 6.34E-10 |
| EMX2OS | 3.495755208 | 1.529055717 | -1.192963186 | 6.79E-06 | 3.46E-05 |
| ANXA3 | 14.44247188 | 29.51845346 | 1.031299452 | 1.46E-08 | 1.73E-07 |
| FGFBP2 | 0.697326563 | 3.594227697 | 2.365775465 | 0.006628758 | 0.01368077 |
| AC012307.1 | 9.23695 | 2.16852029 | -2.090705617 | 3.42E-10 | 7.24E-09 |
| CCL19 | 18.09871042 | 57.94061031 | 1.678687976 | 0.0012808 | 0.003254942 |
| CSPG4 | 11.46211042 | 22.94218068 | 1.001129828 | 6.95E-11 | 1.86E-09 |
| SLC16A1 | 24.61675729 | 54.08334106 | 1.135543547 | 4.99E-12 | 2.02E-10 |
| ACTC1 | 17.4807901 | 43.38801771 | 1.311526281 | 4.66E-09 | 6.53E-08 |
| AP006621.4 | 1.463491667 | 0.687723833 | -1.089513284 | 4.16E-09 | 5.97E-08 |
| AL139349.1 | 3.869153125 | 1.889677778 | -1.033877574 | 6.26E-13 | 3.70E-11 |
| TCAP | 16.78511198 | 2.119701127 | -2.985249394 | 6.18E-09 | 8.30E-08 |
| CXCL12 | 9.502569271 | 25.56952512 | 1.428035825 | 3.05E-07 | 2.35E-06 |
| IGLV1-41 | 2.686938542 | 6.340055717 | 1.238532196 | 0.027086927 | 0.045871061 |
| C11orf96 | 20.99437865 | 47.19584541 | 1.168656776 | 0.000314094 | 0.000960824 |
| LINC00973 | 1.076621875 | 2.529368438 | 1.232265557 | 1.23E-08 | 1.50E-07 |
| PRR16 | 2.473741146 | 5.499056361 | 1.15248953 | 9.60E-10 | 1.74E-08 |
| AL359715.1 | 1.537420833 | 0.759958293 | -1.016519973 | 5.85E-14 | 5.34E-12 |
| SPHK1 | 17.20690104 | 38.05873124 | 1.145240176 | 5.06E-13 | 3.08E-11 |
| ACTG2 | 98.83174271 | 304.0707116 | 1.621360476 | 3.80E-06 | 2.08E-05 |
| LINC01711 | 1.281223958 | 3.006578905 | 1.23060014 | 1.23E-06 | 7.84E-06 |
| GAST | 0.638882813 | 1.347874879 | 1.077063347 | 0.002690828 | 0.006228358 |
| AC008870.5 | 1.813471875 | 0.65121562 | -1.477547161 | 5.70E-06 | 2.98E-05 |
| PDE2A-AS2 | 0.870598958 | 1.959438164 | 1.170359846 | 0.001686061 | 0.004142943 |
| AL359715.2 | 1.661598958 | 0.784861192 | -1.082062786 | 6.31E-15 | 9.20E-13 |
| MTND4P20 | 1.538850521 | 0.576244444 | -1.417100258 | 6.96E-05 | 0.000259941 |
| AOX1 | 1.179440625 | 2.77242818 | 1.233047295 | 4.75E-09 | 6.63E-08 |
| SIGLEC6 | 1.49126875 | 3.51514058 | 1.23704211 | 0.017651477 | 0.031819443 |
| PPBP | 1.630684375 | 3.759079227 | 1.204901752 | 0.000937603 | 0.002474453 |
| ABCA10 | 3.478611458 | 1.658092754 | -1.068986834 | 9.93E-06 | 4.77E-05 |
| MIR548AN | 5.126044271 | 1.990095813 | -1.365008047 | 7.13E-11 | 1.89E-09 |
| AADACP1 | 1.327158333 | 2.692795008 | 1.020763909 | 2.02E-05 | 8.81E-05 |
| MYOSLID | 0.628508854 | 2.961353301 | 2.236251646 | 1.00E-08 | 1.26E-07 |
| MFAP5 | 6.300048958 | 16.45079919 | 1.384722728 | 3.80E-10 | 7.90E-09 |
| SLC10A1 | 4.483452604 | 0.582813688 | -2.943503482 | 3.96E-05 | 0.000159974 |
| SPOCD1 | 21.13894479 | 9.796455878 | -1.109571546 | 0.002328777 | 0.005492362 |
| KIF26A | 1.086675521 | 2.523538486 | 1.21552687 | 4.59E-08 | 4.57E-07 |
| TBC1D3L | 10.31275625 | 4.526530596 | -1.187952357 | 8.41E-19 | 9.81E-16 |
| AL354919.2 | 2.150844271 | 0.839145411 | -1.357910338 | 3.70E-06 | 2.04E-05 |
| STK32A-AS1 | 4.662314583 | 1.192306924 | -1.967290689 | 2.97E-06 | 1.67E-05 |
| CD244 | 2.076315625 | 4.924713527 | 1.246014037 | 0.008010921 | 0.016091872 |
| F13A1 | 14.67353125 | 32.82785958 | 1.161704584 | 6.03E-06 | 3.12E-05 |
| AL033527.2 | 1.507819792 | 0.617093398 | -1.28890325 | 1.71E-14 | 2.06E-12 |
| MIR6793 | 1.388907813 | 0.66925153 | -1.053330408 | 7.27E-07 | 4.99E-06 |
| UCA1 | 65.56768333 | 29.63838035 | -1.145518311 | 4.92E-05 | 0.000192905 |
| AC010378.1 | 23.58457865 | 11.03382029 | -1.095911438 | 5.46E-06 | 2.87E-05 |
| NNMT | 80.63005208 | 163.6808573 | 1.021496048 | 1.80E-09 | 2.95E-08 |
| ANGPTL1 | 1.921608854 | 4.829655072 | 1.329605454 | 6.10E-06 | 3.15E-05 |
| NGF | 1.727048958 | 3.874087923 | 1.165547715 | 6.67E-08 | 6.37E-07 |
| AL135999.3 | 14.25864896 | 5.947332689 | -1.261522603 | 5.20E-10 | 1.03E-08 |
| AQP5 | 1.364923438 | 3.698946216 | 1.438294295 | 0.004877459 | 0.01046461 |
| SH3PXD2A-AS1 | 2.845301563 | 8.765052979 | 1.623181245 | 4.63E-06 | 2.47E-05 |
| SRPX | 17.31468333 | 35.14084396 | 1.021152838 | 4.96E-11 | 1.40E-09 |
| RPL37P1 | 5.634764583 | 2.26005942 | -1.317994633 | 3.82E-19 | 5.20E-16 |
| HDAC9 | 0.865786458 | 1.842102738 | 1.089270385 | 1.87E-11 | 6.17E-10 |
| DKK1 | 27.35765156 | 66.87122093 | 1.289441067 | 5.71E-09 | 7.78E-08 |
| CD177 | 5.529140104 | 14.14055781 | 1.354711998 | 0.025240184 | 0.043240505 |
| IGFBP6 | 56.02919583 | 133.6131654 | 1.253811477 | 6.22E-08 | 5.99E-07 |
| ABI3BP | 2.129403646 | 4.444261192 | 1.061494157 | 4.61E-05 | 0.00018279 |
| BCL2A1 | 9.318332813 | 19.45119179 | 1.061714789 | 1.00E-07 | 9.05E-07 |
| KRT84 | 0.120454167 | 2.465479066 | 4.355311799 | 0.006899528 | 0.014178927 |
| GCNA | 1.518171354 | 0.756625121 | -1.004684054 | 5.30E-11 | 1.47E-09 |
| AL356740.1 | 2.687672396 | 1.162830435 | -1.20871656 | 8.20E-13 | 4.59E-11 |
| COL17A1 | 44.72707656 | 109.1349723 | 1.286893118 | 0.001024657 | 0.002669243 |
| KRT31 | 3.818258333 | 22.90936345 | 2.584950753 | 0.019912098 | 0.035327203 |
| AC092490.1 | 1.049238021 | 2.770477939 | 1.400792887 | 0.000142047 | 0.000484765 |
| ADGRG3 | 0.825619271 | 1.818465539 | 1.139173036 | 8.73E-09 | 1.12E-07 |
| FAM166C | 1.551772917 | 0.759224799 | -1.031318431 | 0.000115868 | 0.000406909 |
| KLK13 | 2.657733854 | 7.434316264 | 1.484003423 | 0.001352638 | 0.003416232 |
| ARSI | 5.596125 | 12.0004087 | 1.100583447 | 1.28E-13 | 9.88E-12 |
| PI15 | 1.471996875 | 3.028386634 | 1.040774798 | 8.44E-07 | 5.67E-06 |
| LINC00930 | 7.840882813 | 2.545249919 | -1.623208776 | 2.05E-16 | 5.13E-14 |
| TBX3 | 129.9770521 | 58.93242013 | -1.141123514 | 6.38E-15 | 9.21E-13 |
| AL139246.4 | 2.067841146 | 0.753399034 | -1.456639272 | 1.34E-07 | 1.16E-06 |
| AL691482.3 | 19.36828333 | 7.416476651 | -1.384890217 | 1.11E-16 | 3.13E-14 |
| CCDC190 | 1.198954688 | 5.180759259 | 2.11138641 | 0.008241691 | 0.016498544 |
| KRT6B | 105.1763448 | 379.344423 | 1.850698063 | 1.39E-07 | 1.20E-06 |
| TLR10 | 0.555160938 | 1.571126892 | 1.50082174 | 0.000420366 | 0.001232436 |
| CAVIN2 | 7.085495833 | 14.81992399 | 1.064597329 | 9.91E-06 | 4.76E-05 |
| AL450384.2 | 7.33824375 | 2.916532045 | -1.331180901 | 1.90E-19 | 3.45E-16 |
| S100A7 | 586.1189391 | 1273.841107 | 1.119919973 | 0.01065009 | 0.020603705 |
| MCF2L-AS1 | 12.90348958 | 6.434879871 | -1.003776156 | 1.30E-13 | 9.92E-12 |
| CCR2 | 1.072446354 | 2.428413043 | 1.179108344 | 0.000957296 | 0.002516291 |
| EFEMP1 | 21.81211094 | 81.24659275 | 1.897177914 | 3.62E-15 | 5.68E-13 |
| CPXM2 | 7.095444792 | 14.93046473 | 1.073294042 | 4.14E-06 | 2.24E-05 |
| MELTF | 8.811659375 | 19.21915765 | 1.125059474 | 3.76E-10 | 7.83E-09 |
| AL121761.1 | 0.9965625 | 3.024567472 | 1.601696652 | 0.011234309 | 0.021563691 |
| AL139246.1 | 2.141684375 | 0.895444444 | -1.258070051 | 7.77E-07 | 5.28E-06 |
| SORBS1 | 8.388247917 | 18.0992649 | 1.109489697 | 0.001360662 | 0.003433311 |
| VAT1L | 0.909535417 | 1.876304026 | 1.044691891 | 0.001548109 | 0.003844483 |
| PILRB | 4.050989063 | 1.928326892 | -1.07092455 | 2.03E-09 | 3.26E-08 |
| NT5E | 15.87291302 | 39.50904605 | 1.315616095 | 1.85E-09 | 3.00E-08 |
| PRSS43P | 1.707905208 | 0.677289855 | -1.334382614 | 1.53E-05 | 6.94E-05 |
| PPFIBP2 | 34.84632188 | 16.94424911 | -1.040210679 | 3.29E-15 | 5.27E-13 |
| AC053503.3 | 1.472930208 | 4.311172303 | 1.54939115 | 0.001261515 | 0.003210432 |
| CAV2 | 22.7570224 | 49.53381965 | 1.122102071 | 5.22E-15 | 7.82E-13 |
| AC018978.1 | 1.202117708 | 2.454594203 | 1.029906368 | 7.50E-09 | 9.86E-08 |
| CR2 | 1.294223438 | 11.1880037 | 3.111794023 | 0.001266871 | 0.003222555 |
| S100A2 | 1113.583946 | 2286.909645 | 1.038189048 | 0.007578628 | 0.015355752 |
| CDH26 | 13.66606719 | 6.684768116 | -1.031648703 | 1.23E-06 | 7.83E-06 |
| MT1L | 10.58390313 | 34.52540145 | 1.705786428 | 3.55E-11 | 1.05E-09 |
| TGFB2 | 2.017980729 | 4.316814171 | 1.097054592 | 1.35E-07 | 1.17E-06 |
| PAQR6 | 11.59542083 | 5.084901449 | -1.189263462 | 6.82E-14 | 5.95E-12 |
| B3GAT1 | 2.41249375 | 0.889393881 | -1.439630821 | 0.002106981 | 0.005029578 |
| IGFL2 | 3.975156771 | 13.53847472 | 1.767981547 | 0.001329204 | 0.003364857 |
| HSPA4L | 2.502071354 | 5.9109657 | 1.240270916 | 1.91E-11 | 6.24E-10 |
| AC005009.1 | 2.148365625 | 0.781461997 | -1.458991922 | 2.90E-09 | 4.43E-08 |
| AC021146.12 | 4.207620833 | 1.830816586 | -1.200517436 | 7.89E-05 | 0.000290623 |
| ABCC9 | 0.757923958 | 1.832194042 | 1.273447286 | 2.21E-08 | 2.46E-07 |
| HES6 | 41.66598906 | 17.03817279 | -1.290099601 | 7.43E-06 | 3.73E-05 |
| TCL1A | 0.695744271 | 5.727614493 | 3.041305364 | 0.010986705 | 0.021160588 |
| ELF5 | 13.89219948 | 6.865831884 | -1.016768595 | 7.42E-05 | 0.000275236 |
| IGHM | 160.2019328 | 453.1242021 | 1.500014996 | 0.018119675 | 0.032530516 |
| LINC02544 | 1.485467708 | 3.81071256 | 1.359143547 | 4.23E-07 | 3.14E-06 |
| PLN | 5.342958333 | 15.910557 | 1.574273672 | 1.44E-07 | 1.23E-06 |
| GYPC | 7.881916667 | 16.10000548 | 1.030442777 | 1.93E-08 | 2.20E-07 |
| KRTAP5-9 | 8.533346354 | 3.54021723 | -1.269273718 | 1.84E-09 | 3.00E-08 |
| MT1M | 11.40947552 | 29.00764911 | 1.346200905 | 3.83E-08 | 3.92E-07 |
| AC091182.2 | 1.762618229 | 3.583570692 | 1.023677784 | 0.000293494 | 0.000906815 |
| TM4SF1 | 107.2742432 | 234.8101338 | 1.130190949 | 2.71E-13 | 1.81E-11 |
| ASB2 | 2.190666667 | 4.78565942 | 1.127347749 | 2.15E-06 | 1.26E-05 |
| CAPN9 | 5.142340625 | 2.547269082 | -1.013473809 | 0.000167826 | 0.000559867 |
| TRBV7-2 | 2.21745625 | 4.54807343 | 1.036349906 | 0.005593112 | 0.011789305 |
| LINC00942 | 5.321019792 | 12.28642834 | 1.207290911 | 0.022389668 | 0.03906893 |
| TPSD1 | 1.252504688 | 2.565798229 | 1.03459172 | 0.0017233 | 0.004224271 |
| OGN | 1.850038542 | 4.516099517 | 1.287521951 | 8.45E-06 | 4.16E-05 |
| KRT6C | 21.19914635 | 83.10078631 | 1.970855956 | 6.09E-07 | 4.30E-06 |
| AC010998.3 | 5.054059896 | 2.05667971 | -1.297125624 | 8.69E-07 | 5.81E-06 |
| HRH3 | 2.145142188 | 0.647762158 | -1.727537184 | 1.78E-09 | 2.93E-08 |
| TNFRSF14-AS1 | 3.7087875 | 1.814836554 | -1.031107986 | 7.08E-05 | 0.000263998 |
| CASQ2 | 3.95593125 | 10.17703382 | 1.363227878 | 2.41E-06 | 1.40E-05 |
| DSG3 | 25.45587344 | 112.5392837 | 2.144358213 | 4.33E-09 | 6.15E-08 |
| PTHLH | 13.92955625 | 42.43244187 | 1.607018405 | 3.12E-11 | 9.45E-10 |
| CES1 | 36.79704271 | 150.1541995 | 2.028783095 | 9.97E-05 | 0.000356067 |
| AC136475.9 | 0.333359896 | 1.714889211 | 2.362962916 | 0.006917556 | 0.014210329 |
| SNX31 | 103.2666865 | 44.17738309 | -1.224995054 | 1.93E-09 | 3.11E-08 |
| IGF2BP2 | 11.83566979 | 26.08896296 | 1.140298246 | 3.78E-08 | 3.88E-07 |
| HMGCS2 | 457.3919516 | 117.5951391 | -1.959602553 | 2.53E-10 | 5.58E-09 |
| SEMG1 | 4.527839063 | 0.345915137 | -3.710332629 | 0.002156461 | 0.005129357 |
| WFDC21P | 83.21743958 | 38.93429469 | -1.095844409 | 0.023169078 | 0.040198074 |
| ERVE-1 | 9.85050625 | 4.339809018 | -1.182566316 | 1.16E-10 | 2.88E-09 |
| CARMN | 1.210851563 | 2.540875362 | 1.069303592 | 0.021144869 | 0.03719667 |
| AC005224.3 | 0.522494792 | 1.716423833 | 1.715917277 | 1.97E-08 | 2.24E-07 |
| TNIP3 | 0.579641146 | 1.482268116 | 1.354574516 | 3.77E-06 | 2.07E-05 |
| CA4 | 8.029546875 | 2.886981643 | -1.475756642 | 5.90E-07 | 4.18E-06 |
| RNU4-78P | 3.471546875 | 1.603953462 | -1.113946368 | 4.81E-12 | 1.96E-10 |
| TMEM145 | 2.220277604 | 1.04677037 | -1.084795076 | 0.000203876 | 0.000663848 |
| SYNPO2 | 6.403916667 | 16.66106103 | 1.37945384 | 0.000182272 | 0.000601806 |
| AC006042.1 | 11.22119427 | 5.524100805 | -1.022414678 | 1.83E-14 | 2.20E-12 |
| SPINK4 | 23.91961979 | 9.394541063 | -1.348299867 | 0.0002041 | 0.000664443 |
| VNN1 | 1.606282292 | 3.227661514 | 1.006763833 | 3.43E-07 | 2.60E-06 |
| AL844908.1 | 1.140220833 | 2.589727053 | 1.183486785 | 0.001849054 | 0.004494649 |
| SPP1 | 238.2652552 | 620.9592089 | 1.381929913 | 5.72E-06 | 2.98E-05 |
| SPINK6 | 2.329061979 | 13.32365813 | 2.516169305 | 6.31E-06 | 3.24E-05 |
| RN7SL3 | 12.73739896 | 29.83212882 | 1.22779623 | 0.009080568 | 0.017966316 |
| CLEC1A | 1.829318229 | 3.88740161 | 1.087500092 | 0.002902812 | 0.006653928 |
| PNCK | 25.17034323 | 9.721039291 | -1.372542422 | 1.30E-07 | 1.13E-06 |
| CNTNAP2 | 0.650846354 | 1.676300322 | 1.364891732 | 0.002557719 | 0.005944703 |
| VASH2 | 3.753598958 | 1.520047826 | -1.304157801 | 1.03E-07 | 9.30E-07 |
| HS6ST3 | 4.337475521 | 1.628862158 | -1.412991094 | 6.78E-07 | 4.71E-06 |
| DAB1 | 4.631607813 | 1.606572625 | -1.527526897 | 1.46E-05 | 6.64E-05 |
| CGA | 0.7169625 | 9.982908696 | 3.799490664 | 0.021548445 | 0.037812866 |
| VNN2 | 1.480341146 | 3.218485507 | 1.120452288 | 5.19E-05 | 0.000201932 |
| PCDH7 | 4.919395833 | 10.09050097 | 1.036444753 | 9.80E-11 | 2.49E-09 |
| CYCSP6 | 2.255441667 | 4.642118519 | 1.041373383 | 0.003233275 | 0.007307774 |
| SPON1 | 10.17677656 | 25.01958873 | 1.297777407 | 2.63E-08 | 2.85E-07 |
| FNDC1 | 6.666176042 | 13.33993688 | 1.000820518 | 4.97E-08 | 4.92E-07 |
| TTC9B | 1.660859375 | 0.407436554 | -2.0272826 | 1.08E-05 | 5.10E-05 |
| LINC01668 | 9.970796354 | 3.410772464 | -1.547610221 | 1.07E-10 | 2.70E-09 |
| CKS1BP1 | 1.726608854 | 3.493230918 | 1.016620722 | 1.71E-07 | 1.43E-06 |
| AC008739.2 | 3.618382813 | 1.631898712 | -1.148793533 | 1.32E-11 | 4.61E-10 |
| TWIST2 | 2.622377604 | 7.00316409 | 1.417131453 | 7.14E-10 | 1.35E-08 |
| KCNJ5 | 1.088688021 | 2.348047021 | 1.108870711 | 5.53E-06 | 2.90E-05 |
| KRTAP5-8 | 2.146258854 | 0.847096779 | -1.341225376 | 7.18E-10 | 1.35E-08 |
| SERPINB7 | 1.86940625 | 13.7590934 | 2.879733385 | 1.84E-11 | 6.11E-10 |
| AL592211.1 | 3.232422396 | 1.563580837 | -1.047761925 | 4.59E-17 | 1.63E-14 |
| LAMC2 | 109.2222927 | 230.576601 | 1.077978769 | 6.18E-06 | 3.19E-05 |
| MTRNR2L12 | 7.679050521 | 3.797903221 | -1.015724797 | 0.000555106 | 0.001562241 |
| AL008627.1 | 1.758377604 | 0.571380032 | -1.621722391 | 0.012442949 | 0.023564944 |
| HAND1 | 0.3645625 | 1.602643478 | 2.136215447 | 5.54E-05 | 0.000213695 |
| PDE1A | 1.056904688 | 2.178298712 | 1.043356526 | 6.59E-06 | 3.36E-05 |
| GAS2 | 2.440697917 | 0.869191304 | -1.489548098 | 2.61E-06 | 1.50E-05 |
| NAPSB | 6.419718229 | 15.16586135 | 1.240245557 | 0.000906691 | 0.002406099 |
| PDLIM3 | 6.500538542 | 13.53094879 | 1.057631856 | 1.26E-09 | 2.19E-08 |
| HSPB6 | 5.697069792 | 14.68163317 | 1.365720476 | 2.46E-05 | 0.000104984 |
| STK19B | 1.523378125 | 3.074725604 | 1.013183582 | 0.0119657 | 0.022777391 |
| SFRP1 | 6.262553125 | 14.68308937 | 1.229332706 | 5.53E-06 | 2.90E-05 |
| MT1E | 54.73238958 | 139.6008979 | 1.350841471 | 5.94E-09 | 8.02E-08 |
| ALDH1A2 | 13.14723646 | 3.718211272 | -1.822078925 | 0.000128973 | 0.000446685 |
| CD1C | 1.513574479 | 3.701155233 | 1.290015978 | 0.00275011 | 0.006352429 |
| CYP4Z2P | 4.762263542 | 1.890858937 | -1.332605722 | 2.40E-13 | 1.66E-11 |
| PSORS1C3 | 18.6187224 | 7.568465217 | -1.298681404 | 9.86E-08 | 8.93E-07 |
| HPSE2 | 1.579389583 | 3.969313688 | 1.3295225 | 9.23E-06 | 4.47E-05 |
| AP001347.2 | 3.256548958 | 1.477653945 | -1.140035475 | 2.92E-05 | 0.000121964 |
| MIR6784 | 6.228327083 | 2.625592432 | -1.246201725 | 1.79E-06 | 1.08E-05 |
| CTSE | 84.88699063 | 35.39478744 | -1.262006559 | 3.23E-09 | 4.84E-08 |
| KIAA1755 | 1.115313021 | 2.246915137 | 1.010496967 | 2.39E-08 | 2.63E-07 |
| VWA5B1 | 2.282878646 | 0.881911755 | -1.372147959 | 1.12E-05 | 5.29E-05 |
| CDA | 23.40157188 | 61.61689533 | 1.396720553 | 3.91E-08 | 3.99E-07 |
| MIR200B | 6.063767188 | 2.867179066 | -1.080582355 | 1.76E-07 | 1.47E-06 |
| HOXB3 | 15.52430625 | 7.037267955 | -1.141441447 | 1.36E-10 | 3.33E-09 |
| AL161729.4 | 2.043727083 | 0.999271498 | -1.032253943 | 3.24E-12 | 1.42E-10 |
| SLC2A4 | 1.176377083 | 2.473707085 | 1.072324094 | 0.0003094 | 0.000949489 |
| AC005393.1 | 4.465936458 | 1.608777778 | -1.472997666 | 1.12E-08 | 1.38E-07 |
| AC027348.1 | 3.127521354 | 1.069533494 | -1.548038071 | 2.16E-13 | 1.51E-11 |
| ACOX2 | 1.108408333 | 2.247099356 | 1.019574452 | 1.24E-08 | 1.50E-07 |
| CRYAB | 10.31436094 | 21.50279919 | 1.059870043 | 2.91E-12 | 1.28E-10 |
| LTF | 30.8015349 | 153.2380568 | 2.314700487 | 0.024325047 | 0.041919102 |
| RPSAP52 | 0.509472396 | 1.663156844 | 1.706848343 | 1.66E-09 | 2.77E-08 |
| TPM2 | 157.7050047 | 332.9814588 | 1.078213403 | 1.41E-07 | 1.20E-06 |
| CILP | 4.449179688 | 15.52453108 | 1.802938422 | 6.95E-05 | 0.000259757 |
| CCDC8 | 5.544192708 | 11.55991337 | 1.060081276 | 1.23E-07 | 1.08E-06 |
| MT2A | 561.0346635 | 1215.000737 | 1.114795374 | 1.02E-12 | 5.46E-11 |
| FMO9P | 51.74974063 | 20.56337198 | -1.331474775 | 3.46E-09 | 5.11E-08 |
| MYCL-AS1 | 2.35748125 | 0.986180515 | -1.257322643 | 3.58E-10 | 7.50E-09 |
| AP000553.2 | 2.824591146 | 1.382861192 | -1.030385706 | 2.71E-13 | 1.81E-11 |
| AC068594.1 | 1.441708854 | 0.624478261 | -1.207056594 | 6.98E-08 | 6.61E-07 |
| MOGAT2 | 5.884128646 | 1.968949275 | -1.579402844 | 1.68E-07 | 1.41E-06 |
| AL359881.1 | 3.096517708 | 1.420141546 | -1.124611966 | 2.65E-05 | 0.000111838 |
| AC010275.1 | 1.22794375 | 2.809128986 | 1.193878395 | 0.00034136 | 0.001030657 |
| HKDC1 | 1.396697917 | 2.890765217 | 1.049431418 | 0.001082918 | 0.002803564 |
| BPIFB1 | 37.17095677 | 12.18575233 | -1.608980499 | 0.003322925 | 0.007476867 |
| CAV1 | 75.24426146 | 214.8777742 | 1.513862803 | 3.29E-16 | 7.37E-14 |
| TOX3 | 20.2038849 | 6.46072351 | -1.644865087 | 1.34E-11 | 4.65E-10 |
| CCN5 | 2.320026563 | 7.302355717 | 1.654220625 | 0.00015698 | 0.000528111 |
| MEDAG | 6.1829 | 18.671281 | 1.594465334 | 1.31E-08 | 1.58E-07 |
| AC133041.1 | 9.358167188 | 4.394424477 | -1.090551771 | 4.71E-09 | 6.59E-08 |
| CD52 | 25.56088021 | 57.0992351 | 1.159533902 | 1.00E-05 | 4.81E-05 |
| CMA1 | 0.845975521 | 4.10421095 | 2.278417062 | 0.000113646 | 0.000399793 |
| SULT2B1 | 6.493219271 | 13.15546522 | 1.018656434 | 5.62E-05 | 0.000216496 |
| SPRR2E | 36.43035781 | 86.24812931 | 1.243352001 | 0.000565208 | 0.001586296 |
| MSC | 11.42920885 | 23.91831288 | 1.065390089 | 1.58E-08 | 1.85E-07 |
| AC026803.3 | 2.435806771 | 1.168218841 | -1.060089133 | 7.19E-11 | 1.91E-09 |
| P2RY1 | 2.880507813 | 6.327672786 | 1.135351827 | 0.000117636 | 0.000411966 |
| ALDH1L2 | 1.56480625 | 3.647352979 | 1.220865788 | 1.72E-13 | 1.25E-11 |
| CXCL13 | 37.08978073 | 88.50353784 | 1.254713388 | 5.36E-05 | 0.000207556 |
| VPREB3 | 2.590448438 | 16.52677262 | 2.673531248 | 0.014596012 | 0.027002205 |
| IGHE | 0.921017188 | 3.159556683 | 1.778422164 | 0.012291831 | 0.023320189 |
| BDKRB1 | 1.576436458 | 3.731107407 | 1.242936871 | 2.28E-10 | 5.08E-09 |
| FN1 | 270.2270714 | 585.7603021 | 1.116138212 | 1.88E-10 | 4.36E-09 |
| SPRR2D | 24.15333021 | 86.46056055 | 1.839820071 | 0.00112731 | 0.002904211 |
| TM4SF19 | 0.759914583 | 1.887499356 | 1.312566983 | 1.16E-06 | 7.46E-06 |
| GAS6-DT | 0.870935938 | 2.322133011 | 1.414812103 | 8.94E-10 | 1.64E-08 |
| SERPINB13 | 11.46571979 | 28.79073607 | 1.328277747 | 8.76E-06 | 4.28E-05 |
| PPP1R1B | 26.65704688 | 12.29545266 | -1.116392116 | 0.003024333 | 0.00689772 |
| AL121829.2 | 1.959606771 | 0.945461353 | -1.05147379 | 4.39E-14 | 4.24E-12 |
| HSD17B2 | 18.55738802 | 7.520648631 | -1.303064663 | 1.06E-08 | 1.32E-07 |
| TCHH | 0.657088021 | 2.227470531 | 1.761247799 | 1.62E-05 | 7.29E-05 |
| COPZ2 | 15.12921094 | 30.77063124 | 1.024217294 | 5.03E-13 | 3.07E-11 |
| DSC2 | 15.277125 | 32.61117488 | 1.093993349 | 1.94E-10 | 4.47E-09 |
| TRBV5-6 | 0.666805208 | 1.349888889 | 1.017503384 | 0.004595368 | 0.009936441 |
| CTXND1 | 3.134935417 | 1.156921095 | -1.43814525 | 2.85E-07 | 2.22E-06 |
| IL37 | 3.6935875 | 0.567191626 | -2.703114616 | 1.65E-06 | 1.00E-05 |
| AC110285.2 | 9.260201563 | 3.927931884 | -1.237273685 | 2.32E-12 | 1.07E-10 |
| PLD4 | 1.466136458 | 3.09272029 | 1.076856974 | 0.000622061 | 0.00172803 |
| CD79A | 11.8639526 | 45.05366409 | 1.925059702 | 0.008931001 | 0.017717639 |
| ADAMTSL1 | 0.612236979 | 1.407197585 | 1.20066282 | 2.54E-07 | 2.01E-06 |
| GATA5 | 0.990892188 | 2.216115459 | 1.161233046 | 0.003747243 | 0.008299355 |
| SERPINB2 | 6.487397917 | 29.70976232 | 2.195225226 | 5.17E-12 | 2.07E-10 |
| PID1 | 1.904985938 | 4.851587923 | 1.34867667 | 1.24E-08 | 1.50E-07 |
| FOXC2 | 0.884405208 | 2.864038003 | 1.69527121 | 5.60E-06 | 2.93E-05 |
| SPRR2F | 2.469363021 | 11.7672781 | 2.2525698 | 0.027819649 | 0.046916017 |
| ITGA5 | 41.69138854 | 90.61369791 | 1.119979734 | 6.91E-13 | 3.97E-11 |
| AC018865.2 | 1.476411458 | 0.674495491 | -1.130214135 | 0.001676245 | 0.004123168 |
| FGFR1 | 5.912365104 | 15.32986812 | 1.374538018 | 8.28E-07 | 5.57E-06 |
| HOXB-AS3 | 4.893335938 | 2.014975362 | -1.280056132 | 4.78E-05 | 0.000188247 |
| ZBED2 | 5.736859375 | 14.62917359 | 1.350515214 | 4.27E-10 | 8.72E-09 |
| LINC01356 | 0.156277604 | 2.174275845 | 3.798352035 | 0.00183178 | 0.004454731 |
| SDR16C5 | 4.004204688 | 10.36489936 | 1.372118479 | 0.000168287 | 0.000561288 |
| AL021407.1 | 2.454891146 | 1.218374396 | -1.010701525 | 1.57E-13 | 1.18E-11 |
| ACTA2-AS1 | 0.671830208 | 1.452224155 | 1.112095582 | 0.002197682 | 0.005214973 |
| RTL9 | 0.085836458 | 2.846698712 | 5.051555448 | 9.87E-05 | 0.000353511 |
| TTLL3 | 7.429636979 | 3.566825121 | -1.058651238 | 3.85E-17 | 1.48E-14 |
| PDCD1LG2 | 3.891515104 | 8.237980998 | 1.081958842 | 7.92E-11 | 2.08E-09 |
| CYP2C8 | 1.568447917 | 0.6142438 | -1.352454326 | 5.74E-10 | 1.12E-08 |
| BNC1 | 4.176755729 | 13.44845137 | 1.686985374 | 2.89E-11 | 8.87E-10 |
| CD109 | 7.820329167 | 22.83776409 | 1.546120173 | 2.47E-19 | 3.67E-16 |
| LRTM1 | 2.834482292 | 0.276004026 | -3.360324041 | 0.001378963 | 0.003473783 |
| SYNC | 0.889408854 | 1.805910306 | 1.021807568 | 5.79E-15 | 8.59E-13 |
| AC010329.1 | 6.454554688 | 2.990205958 | -1.110072707 | 1.44E-06 | 8.96E-06 |
| AC012354.2 | 18.8922276 | 5.278894847 | -1.839484988 | 6.62E-09 | 8.80E-08 |
| MEOX1 | 1.729460938 | 3.924300966 | 1.182113262 | 6.57E-05 | 0.000247417 |
| AC112721.1 | 0.3635 | 3.690864734 | 3.343931596 | 1.40E-07 | 1.20E-06 |
| BX119927.1 | 0.352890104 | 2.096953301 | 2.571003854 | 0.000443945 | 0.001290562 |
| AC010329.2 | 2.796298958 | 0.919956844 | -1.603880521 | 3.35E-09 | 4.98E-08 |
| VSNL1 | 4.447077083 | 13.16533027 | 1.565814397 | 1.73E-09 | 2.87E-08 |
| AC112721.2 | 0.645785938 | 4.00065942 | 2.631109885 | 7.70E-07 | 5.24E-06 |
| SLC38A4 | 20.3276125 | 7.376749275 | -1.462383672 | 0.009527231 | 0.018713958 |
| AL021407.2 | 1.862192708 | 0.891851691 | -1.062126653 | 1.80E-12 | 8.59E-11 |
| GSTM2 | 14.71254479 | 4.954189211 | -1.570325933 | 4.07E-06 | 2.21E-05 |
| AC012354.4 | 2.348670833 | 0.776322222 | -1.597117043 | 7.59E-08 | 7.12E-07 |
| PTGDR2 | 1.864628646 | 0.512094525 | -1.864406296 | 2.01E-06 | 1.19E-05 |
| FGF2 | 1.058326563 | 2.916171014 | 1.462290466 | 9.70E-08 | 8.82E-07 |
| ANKRD29 | 0.917196875 | 2.051868277 | 1.161634773 | 3.63E-12 | 1.55E-10 |
| CORO6 | 2.971096875 | 6.075008535 | 1.031890789 | 1.30E-05 | 6.02E-05 |
| MT1H | 3.167413542 | 7.727400805 | 1.28667799 | 0.000198576 | 0.000648402 |
| DCN | 43.33132552 | 91.6901211 | 1.081355933 | 5.93E-08 | 5.75E-07 |
| CD79B | 2.357138542 | 10.08464879 | 2.09705238 | 0.005410908 | 0.011446641 |
| IRF8 | 4.816706771 | 10.36522238 | 1.105632065 | 3.70E-07 | 2.79E-06 |
| SLC5A1 | 0.497646875 | 1.573446699 | 1.660734018 | 0.020643286 | 0.0364124 |
| CCL21 | 25.44539063 | 64.41940773 | 1.340091058 | 6.33E-08 | 6.08E-07 |
| FCER2 | 0.543342188 | 3.761262963 | 2.7912842 | 0.026830455 | 0.045515851 |
| MYLK | 8.626261458 | 18.72479887 | 1.118142873 | 3.09E-09 | 4.66E-08 |
| HSPB7 | 5.610329167 | 16.14175314 | 1.524639953 | 2.76E-07 | 2.15E-06 |
| SLAMF1 | 1.154355729 | 2.663113043 | 1.206025792 | 0.000443021 | 0.001288338 |
| CPXM1 | 24.71375833 | 67.88053945 | 1.457683606 | 4.00E-09 | 5.80E-08 |
| AC008759.2 | 3.346070313 | 1.435324155 | -1.221091169 | 4.20E-12 | 1.75E-10 |
| CFAP251 | 1.563140625 | 3.35779211 | 1.103065338 | 0.009611853 | 0.01885298 |
| ACSM6 | 5.390595313 | 1.903157327 | -1.502049778 | 1.47E-10 | 3.56E-09 |
| AL139288.1 | 9.028058333 | 4.473510145 | -1.013008452 | 1.30E-05 | 6.01E-05 |
| SH2D5 | 0.819416146 | 2.651862319 | 1.694337649 | 6.25E-08 | 6.00E-07 |
| CRH | 212.9720698 | 47.47213494 | -2.165511403 | 1.10E-07 | 9.78E-07 |
| NUDT11 | 1.636220313 | 4.12613752 | 1.334424889 | 6.94E-08 | 6.58E-07 |
| CCBE1 | 0.999105729 | 2.75853591 | 1.465193498 | 7.97E-05 | 0.000292809 |
| KRT1 | 40.06566667 | 155.921409 | 1.960380645 | 0.003053111 | 0.006954498 |
| SSTR5-AS1 | 1.637315625 | 0.453161031 | -1.853236749 | 0.00034217 | 0.001032583 |
| ARSJ | 1.514773958 | 4.859343639 | 1.681658935 | 5.63E-17 | 1.88E-14 |
| CPA4 | 5.346688021 | 15.6694781 | 1.551239726 | 9.26E-10 | 1.69E-08 |
| KRT17P3 | 1.426010417 | 5.581122222 | 1.96857072 | 0.000975849 | 0.002560486 |
| SBK1 | 23.60889583 | 9.527366023 | -1.309181249 | 2.11E-11 | 6.75E-10 |
| FAM3D | 20.16257708 | 9.673366184 | -1.059590131 | 2.99E-06 | 1.69E-05 |
| TREM1 | 3.226673958 | 6.916780998 | 1.100052971 | 9.56E-08 | 8.71E-07 |
| AL137800.1 | 0.468382813 | 1.917744122 | 2.033650198 | 0.000274538 | 0.000855696 |
| KRTAP2-3 | 0.391339063 | 2.16090467 | 2.465144399 | 0.010081822 | 0.019623167 |
| HDC | 0.738910417 | 1.559439614 | 1.077556316 | 0.001480176 | 0.003697731 |
| CALML3 | 3.110852083 | 7.138737359 | 1.198359126 | 0.01908285 | 0.034020244 |
| SPRR2G | 13.38601875 | 39.58818921 | 1.564343139 | 0.008729342 | 0.017372476 |
| AARD | 0.521044271 | 1.512270853 | 1.537238693 | 6.56E-05 | 0.000247153 |
| AC008982.2 | 3.446910938 | 1.601024638 | -1.106308513 | 0.000248787 | 0.000786549 |
| REG4 | 3.176015625 | 0.980372464 | -1.695816142 | 3.60E-06 | 1.98E-05 |
| MIR429 | 31.93333698 | 12.92414171 | -1.304994846 | 6.57E-13 | 3.83E-11 |
| FGF7 | 2.666426563 | 7.909081159 | 1.568602504 | 1.52E-08 | 1.79E-07 |
| AC078880.3 | 4.54784375 | 1.131486634 | -2.006963145 | 4.78E-10 | 9.57E-09 |
| LGR5 | 2.508891667 | 1.109309018 | -1.177388867 | 2.25E-06 | 1.32E-05 |
| TEX45 | 2.821335417 | 1.173894847 | -1.265075009 | 8.04E-16 | 1.53E-13 |
| PCOLCE2 | 2.725059896 | 5.774917874 | 1.08351249 | 1.99E-07 | 1.63E-06 |
| RHCG | 23.10965833 | 112.0936435 | 2.278136634 | 0.000225164 | 0.000721795 |
| LINC01977 | 1.418915625 | 0.699262963 | -1.020881804 | 4.42E-13 | 2.74E-11 |
| MIR200A | 11.17471875 | 4.889087601 | -1.192601361 | 2.74E-10 | 5.95E-09 |
| P2RX1 | 2.87740625 | 6.679431562 | 1.214956407 | 7.24E-07 | 4.98E-06 |
| NTRK2 | 1.462718229 | 2.948020934 | 1.011094887 | 0.020473638 | 0.036171982 |
| PCP2 | 5.153546875 | 2.430300805 | -1.084430803 | 3.44E-16 | 7.59E-14 |
| ZNF334 | 4.195286979 | 2.006392432 | -1.064165691 | 0.007797121 | 0.015714583 |
| TPSB2 | 11.6813375 | 24.13636264 | 1.047002807 | 0.000155346 | 0.000523474 |
| HTRA4 | 0.63408125 | 2.21888277 | 1.807093826 | 0.002265346 | 0.005358244 |
| PCSK9 | 2.649905208 | 6.573729952 | 1.310771439 | 1.82E-09 | 2.97E-08 |
| CASQ1 | 21.13811094 | 6.465335105 | -1.709049399 | 3.32E-12 | 1.44E-10 |
| FLNC | 10.64654323 | 27.73303285 | 1.381220311 | 8.91E-11 | 2.29E-09 |
| PAPPA | 0.667188021 | 1.821107407 | 1.448650723 | 1.88E-06 | 1.12E-05 |
| HPCA | 1.610340104 | 0.678481481 | -1.246984074 | 6.04E-07 | 4.27E-06 |
| LINC02154 | 4.047529688 | 13.51612319 | 1.739567837 | 0.000506176 | 0.001440974 |
| PGLYRP3 | 3.709044792 | 13.30849533 | 1.843227873 | 0.014884154 | 0.027474165 |
| AP005432.2 | 69.37645104 | 24.30529662 | -1.513175301 | 0.00011742 | 0.000411387 |
| LINC00709 | 2.989552083 | 0.478053623 | -2.644684986 | 5.81E-11 | 1.59E-09 |
| LINC01615 | 2.76086875 | 5.781980837 | 1.066441523 | 3.70E-09 | 5.40E-08 |
| AL158847.1 | 5.495925 | 1.3560219 | -2.018981838 | 2.77E-06 | 1.58E-05 |
| TMEM45A | 25.90278854 | 52.03196651 | 1.006290816 | 2.57E-08 | 2.80E-07 |
| ARHGEF4 | 3.895178646 | 8.166744928 | 1.068071672 | 1.44E-05 | 6.57E-05 |
| ZNF114 | 2.745176563 | 6.167508857 | 1.16778894 | 0.000248105 | 0.000784548 |
| TSPAN8 | 14.2995776 | 4.690549436 | -1.608143701 | 0.000169965 | 0.000566309 |
| SPAG17 | 2.7651625 | 1.314774718 | -1.072548645 | 9.43E-07 | 6.23E-06 |
| STEAP4 | 2.659817188 | 7.192563768 | 1.435179016 | 4.47E-11 | 1.28E-09 |
| KIF1A | 2.363585417 | 1.133891948 | -1.059693834 | 0.004263599 | 0.009289206 |
| FGFBP1 | 43.91834323 | 197.6202103 | 2.16983496 | 1.84E-10 | 4.28E-09 |
| C2orf66 | 2.659532813 | 1.134500805 | -1.229115204 | 6.40E-09 | 8.57E-08 |
| ODAPH | 1.443456771 | 3.833859098 | 1.409269414 | 0.000435123 | 0.001268078 |
| ALPK2 | 0.924351042 | 2.146878583 | 1.215727847 | 3.41E-06 | 1.89E-05 |
| GKN1 | 5.554027604 | 1.934704831 | -1.52142087 | 0.001159165 | 0.002977818 |
| AATBC | 23.91342344 | 9.933003704 | -1.267518726 | 8.31E-14 | 7.00E-12 |
| LGALS7B | 19.08178698 | 65.107443 | 1.770626196 | 0.001135976 | 0.002924228 |
| AC099518.2 | 3.723231771 | 1.607926731 | -1.211353758 | 2.30E-10 | 5.13E-09 |
| SLC6A14 | 3.882688021 | 9.857757005 | 1.344203629 | 0.012158105 | 0.023089789 |
| AP001574.1 | 2.410896354 | 1.045298873 | -1.205654132 | 2.91E-08 | 3.11E-07 |
| MSLN | 15.07389427 | 40.93287375 | 1.441207778 | 1.25E-06 | 7.94E-06 |
| RHOT1P1 | 1.859701563 | 0.77134525 | -1.26962247 | 5.55E-06 | 2.91E-05 |
| CCDC198 | 3.817730208 | 1.481433977 | -1.365720822 | 4.82E-07 | 3.52E-06 |
| HOXD11 | 1.256194271 | 2.570461353 | 1.032967726 | 2.26E-07 | 1.82E-06 |
| FOXN4 | 2.255717188 | 0.216347987 | -3.382160597 | 0.000514329 | 0.001460578 |
| SYT12 | 1.800688542 | 4.365225282 | 1.277507446 | 0.000156161 | 0.000525787 |
| MIR7152 | 6.165757292 | 1.946999195 | -1.663025813 | 4.56E-08 | 4.54E-07 |
| TENT5B | 7.364216667 | 16.54941417 | 1.16817617 | 8.19E-10 | 1.52E-08 |
| PDZD3 | 2.044507813 | 0.785155556 | -1.380703161 | 4.02E-09 | 5.82E-08 |
| MUC2 | 8.90431875 | 3.894539936 | -1.193052326 | 3.72E-05 | 0.000150859 |
| SEMA3A | 1.514571875 | 3.895921739 | 1.363054653 | 1.43E-11 | 4.93E-10 |
| SFRP2 | 137.5288417 | 299.9211986 | 1.124849293 | 8.33E-10 | 1.54E-08 |
| NFE4 | 0.936151042 | 3.476423188 | 1.892790491 | 0.001227331 | 0.003130745 |
| RGMA | 1.032601042 | 2.172500805 | 1.073073753 | 8.31E-09 | 1.07E-07 |
| GDF7 | 3.554125 | 1.433574396 | -1.309877646 | 2.69E-13 | 1.80E-11 |
| CLIC3 | 115.3567229 | 267.0318034 | 1.210909491 | 0.000100924 | 0.000359927 |
| AC010643.1 | 1.850479167 | 0.810493398 | -1.191026555 | 5.74E-05 | 0.00022041 |
| SMOC2 | 14.17048021 | 31.74907923 | 1.163826103 | 0.000845254 | 0.002262559 |
| KLK11 | 3.883245833 | 10.78690564 | 1.473946122 | 0.00954354 | 0.018739229 |
| RPL29P19 | 7.336143229 | 17.93424541 | 1.289623334 | 6.95E-06 | 3.52E-05 |
| ANPEP | 13.53683958 | 36.77692061 | 1.44190973 | 1.81E-06 | 1.09E-05 |
| BTBD16 | 76.47312708 | 32.69776264 | -1.225760947 | 1.02E-07 | 9.21E-07 |
| FAM180A | 0.653503125 | 1.566302093 | 1.261096453 | 9.86E-10 | 1.78E-08 |
| KRTAP5-10 | 4.23569375 | 1.472310145 | -1.524516673 | 2.54E-11 | 7.95E-10 |
| CNTN1 | 3.307890625 | 10.09406618 | 1.609524013 | 3.18E-10 | 6.82E-09 |
| MOXD1 | 5.901166667 | 13.28263446 | 1.170469207 | 1.90E-07 | 1.56E-06 |
| AC093849.2 | 0.641439583 | 1.60388599 | 1.322186303 | 4.73E-09 | 6.62E-08 |
| TNFAIP8L3 | 2.858166667 | 7.014677778 | 1.295286789 | 5.02E-14 | 4.74E-12 |
| RPL21P13 | 6.872784896 | 2.507215942 | -1.454808548 | 0.02821887 | 0.047496042 |
| GEM | 13.08533958 | 26.19580129 | 1.001384228 | 4.41E-08 | 4.40E-07 |
| AC010761.4 | 2.56234375 | 1.149554428 | -1.156389257 | 7.82E-14 | 6.65E-12 |
| GZMB | 18.55476198 | 45.1891533 | 1.284187032 | 7.29E-05 | 0.000270885 |
| LCAL1 | 0.955148958 | 1.978723994 | 1.050772741 | 0.007277116 | 0.01483877 |
| HSD11B1 | 3.964747396 | 7.945591948 | 1.002925754 | 0.000173644 | 0.000577623 |
| RHBG | 16.94032396 | 7.118780354 | -1.250759471 | 6.55E-13 | 3.83E-11 |
| LMCD1 | 8.236619271 | 17.48204622 | 1.085749849 | 1.87E-08 | 2.14E-07 |
| NMRAL2P | 3.545286458 | 8.737580354 | 1.301331617 | 0.00023167 | 0.000739402 |
| LINC01541 | 3.140523438 | 0.750368599 | -2.065333675 | 3.59E-08 | 3.74E-07 |
| TBX1 | 37.4344151 | 14.6310525 | -1.355331659 | 4.90E-09 | 6.82E-08 |
| SLC47A1 | 0.687027083 | 1.40950789 | 1.036752676 | 0.003272354 | 0.007379751 |
| PTGFR | 0.909636979 | 1.919708696 | 1.077524597 | 2.47E-07 | 1.97E-06 |
| FOXD1 | 1.496565625 | 3.05444058 | 1.029252632 | 8.46E-05 | 0.000308495 |
| MT1G | 13.6988875 | 30.815843 | 1.169613523 | 2.79E-05 | 0.000117154 |
| RGS20 | 2.411948438 | 4.89649839 | 1.021551344 | 3.81E-08 | 3.90E-07 |
| DSC3 | 32.98468177 | 102.0120989 | 1.628872178 | 5.59E-08 | 5.45E-07 |
| PLIN5 | 9.783557292 | 4.042859742 | -1.27498297 | 2.14E-16 | 5.20E-14 |
| SHD | 1.711988021 | 0.810169726 | -1.079376525 | 0.006396363 | 0.01326319 |
| MS4A8 | 9.260780729 | 0.557712399 | -4.053540574 | 3.75E-07 | 2.82E-06 |
| SLC10A6 | 1.598395833 | 3.809680032 | 1.253045106 | 7.12E-08 | 6.72E-07 |
| PACRG | 3.06300625 | 1.370646055 | -1.160092242 | 6.74E-07 | 4.69E-06 |
| CST6 | 94.45658073 | 256.7658662 | 1.44273021 | 0.000132197 | 0.000455626 |
| SYNM | 12.88981667 | 28.96121562 | 1.167890415 | 1.68E-08 | 1.94E-07 |
| TPSAB1 | 10.34873021 | 22.73255443 | 1.135306048 | 2.09E-06 | 1.23E-05 |
| CTSG | 2.307171354 | 9.372830757 | 2.022359675 | 2.73E-05 | 0.000115022 |
| CDX2 | 3.817045833 | 1.537407568 | -1.311956834 | 1.09E-08 | 1.35E-07 |
| LRRC4 | 0.911392708 | 2.462157166 | 1.433778122 | 1.61E-06 | 9.86E-06 |
| IL20RB | 15.93306771 | 43.35729614 | 1.444250724 | 5.72E-05 | 0.000219805 |
| AC005180.2 | 2.42078125 | 6.038858132 | 1.318803062 | 0.003817955 | 0.008438793 |
| PTGDS | 1.019336979 | 2.207067311 | 1.114499563 | 0.019936341 | 0.035364148 |
| LINC02575 | 8.69668125 | 4.015039452 | -1.115050794 | 0.012612876 | 0.023836978 |
| ENDOU | 0.360726563 | 1.624903865 | 2.171376799 | 4.17E-05 | 0.000166916 |
| CYP3A5 | 13.96037083 | 6.438059259 | -1.116639503 | 5.54E-09 | 7.57E-08 |
| DIO3 | 1.571196875 | 6.361338808 | 2.017466462 | 0.007059162 | 0.014448489 |
| IGKV2D-29 | 13.03175573 | 30.94177037 | 1.247524278 | 0.009911685 | 0.019350369 |
| TMEM163 | 8.201731771 | 4.067400322 | -1.011821572 | 5.83E-09 | 7.92E-08 |
| CGB8 | 0.555692188 | 6.990002738 | 3.652935158 | 7.27E-06 | 3.66E-05 |
| TGM1 | 7.905142188 | 30.74953559 | 1.959701304 | 0.000154534 | 0.000521491 |
| ALX1 | 0.823621354 | 2.149484863 | 1.38393781 | 0.017779593 | 0.032015035 |
| PKP1 | 65.08186094 | 148.3454337 | 1.18863311 | 0.010082675 | 0.019623167 |
| TNC | 34.72150313 | 79.02386892 | 1.186459076 | 1.87E-13 | 1.35E-11 |
| ITGB3 | 2.092782292 | 4.194261997 | 1.002994744 | 1.74E-08 | 2.00E-07 |
| DPT | 3.689492708 | 9.983669726 | 1.436147744 | 1.59E-06 | 9.75E-06 |
| KRT6A | 628.1702349 | 2036.047693 | 1.696543867 | 1.82E-07 | 1.51E-06 |
| CST4 | 11.79107656 | 4.610231884 | -1.354784225 | 0.015252369 | 0.028046008 |
| AC005180.1 | 1.567505729 | 3.898083253 | 1.314294187 | 9.65E-05 | 0.000346753 |
| FAP | 4.146373958 | 8.721749436 | 1.072767307 | 8.76E-11 | 2.26E-09 |
| KRT7-AS | 31.15395052 | 14.77783994 | -1.07597971 | 1.27E-13 | 9.88E-12 |
| RAMP1 | 24.31516458 | 60.43965926 | 1.31363917 | 7.92E-09 | 1.03E-07 |
| TMEM51-AS1 | 3.165126563 | 1.450893237 | -1.125321826 | 1.31E-10 | 3.23E-09 |
| HEPHL1 | 2.0474875 | 6.560357166 | 1.679919718 | 0.003417127 | 0.007658364 |
| ACTA2 | 91.86979167 | 198.1526986 | 1.108950153 | 9.39E-07 | 6.21E-06 |
| AC012354.7 | 5.015996875 | 1.466200483 | -1.774454063 | 1.48E-08 | 1.75E-07 |
| BHMT | 61.07264635 | 21.64312609 | -1.496617466 | 6.43E-10 | 1.22E-08 |
| MIR149 | 2.063710938 | 0.912371014 | -1.177548389 | 1.83E-05 | 8.08E-05 |
| OVGP1 | 10.47958906 | 4.579333494 | -1.194372606 | 1.18E-12 | 6.09E-11 |
| ZNF737 | 18.89233594 | 8.005747665 | -1.238693045 | 1.38E-09 | 2.36E-08 |
| PI16 | 2.717983854 | 11.88761063 | 2.128849976 | 0.002043096 | 0.004898736 |
| CASC22 | 2.516894271 | 0.838911594 | -1.585053923 | 1.15E-10 | 2.88E-09 |
| AC010329.5 | 2.811611979 | 0.70684058 | -1.991940734 | 1.48E-07 | 1.26E-06 |
| CEACAM21 | 1.138680208 | 2.68505314 | 1.23758801 | 2.63E-05 | 0.000111265 |
| MANCR | 0.765397396 | 2.729632206 | 1.834425676 | 2.42E-09 | 3.80E-08 |
| ADAMTSL4 | 7.765970313 | 19.9167934 | 1.358747295 | 1.58E-12 | 7.77E-11 |
| F2RL2 | 1.3790625 | 4.702845089 | 1.769845968 | 3.95E-06 | 2.15E-05 |
| AL031668.2 | 5.893347396 | 2.767918196 | -1.090286005 | 0.00035706 | 0.001069609 |
| AL451050.2 | 3.361095313 | 1.656691304 | -1.020626648 | 6.24E-18 | 4.53E-15 |
| UGT1A1 | 6.607182292 | 3.13895153 | -1.073752397 | 3.81E-08 | 3.91E-07 |
| LINC01857 | 1.229881771 | 2.69117649 | 1.129717372 | 3.17E-05 | 0.000131383 |
| AC037198.1 | 1.431920313 | 3.696469243 | 1.368196702 | 5.43E-05 | 0.000210104 |
| GALNT5 | 2.974966667 | 6.3678562 | 1.097934253 | 4.15E-06 | 2.24E-05 |
| SLC7A11 | 5.513392708 | 15.69931192 | 1.509689058 | 0.002125495 | 0.005067103 |
| ASPG | 1.710667188 | 3.608722383 | 1.076929053 | 0.000338564 | 0.00102437 |
| GLI2 | 0.91379375 | 1.879193076 | 1.040172822 | 1.82E-08 | 2.08E-07 |
| GBP6 | 5.570338542 | 14.72617424 | 1.40254576 | 0.000295213 | 0.000911264 |
| PDLIM4 | 14.49708594 | 30.67635572 | 1.081364171 | 1.75E-09 | 2.88E-08 |
| AP002026.1 | 4.183845313 | 2.056761514 | -1.024454993 | 5.15E-10 | 1.02E-08 |
| AC243830.2 | 1.722258854 | 0.798495974 | -1.108944956 | 1.85E-09 | 3.01E-08 |
| DHRS9 | 5.520454167 | 15.35078519 | 1.475453583 | 1.49E-10 | 3.60E-09 |
| CXCL5 | 8.221680208 | 18.66751997 | 1.183025111 | 3.06E-05 | 0.000127391 |
| CYP4F29P | 11.58707656 | 3.691401932 | -1.650275881 | 1.13E-06 | 7.29E-06 |
| TBX4 | 1.268694792 | 3.02995942 | 1.255953429 | 0.007607619 | 0.015400162 |
| NHLH1 | 3.784443229 | 0.351303382 | -3.429291696 | 0.025243247 | 0.043241094 |
| TNNI2 | 157.4238682 | 76.1106029 | -1.048484942 | 0.000111134 | 0.000391886 |
| APCDD1L | 2.220466667 | 7.524414493 | 1.76071641 | 1.68E-11 | 5.68E-10 |
| GLYATL2 | 0.506656771 | 2.162663768 | 2.09372874 | 0.018889926 | 0.033731568 |
| EPGN | 3.175119271 | 7.822828341 | 1.300879521 | 1.73E-06 | 1.05E-05 |
| GATA3-AS1 | 21.37733802 | 8.536996457 | -1.324281729 | 3.18E-14 | 3.28E-12 |
| AXL | 19.26480469 | 45.48044396 | 1.239278778 | 1.65E-10 | 3.91E-09 |
| INSYN1 | 0.731738021 | 1.953491626 | 1.416655944 | 6.35E-06 | 3.26E-05 |
| ADAM23 | 2.677013021 | 6.479703704 | 1.275303689 | 0.000363631 | 0.001086103 |
| IL36RN | 3.010389583 | 7.684469404 | 1.351995446 | 2.75E-05 | 0.000115575 |
| PCP4 | 19.56542083 | 59.56207826 | 1.606087952 | 0.027810262 | 0.046914727 |
| KRT16 | 396.8063354 | 892.8053448 | 1.169910604 | 0.001458152 | 0.003649964 |
| FDCSP | 15.53852708 | 297.3627047 | 4.258302055 | 0.000190642 | 0.000625623 |
| AL645608.6 | 2.831173438 | 1.257109018 | -1.171290364 | 9.07E-06 | 4.40E-05 |
| FST | 26.78676354 | 56.80944895 | 1.084610628 | 0.003063045 | 0.006970317 |
| PI3 | 589.5779385 | 2420.259977 | 2.037407579 | 0.000830834 | 0.002232684 |
| HNF1B | 12.22528073 | 5.974944283 | -1.032870429 | 4.96E-09 | 6.88E-08 |
| AP001628.2 | 3.481733854 | 1.073495813 | -1.697489362 | 4.35E-09 | 6.17E-08 |
| PM20D1 | 120.6394781 | 51.20121272 | -1.236452206 | 1.30E-06 | 8.23E-06 |
| BDKRB2 | 4.691336458 | 9.678598229 | 1.044799141 | 1.91E-12 | 8.99E-11 |
| CD22 | 0.972909896 | 5.36773285 | 2.463934767 | 0.000130575 | 0.000450988 |
| AP000553.6 | 9.318714583 | 4.659210306 | -1.000045512 | 1.81E-10 | 4.22E-09 |
| DACT3 | 2.722557292 | 5.46807971 | 1.006071865 | 1.63E-06 | 9.95E-06 |
| PLAAT2 | 9.489354167 | 21.38228857 | 1.172034467 | 0.001957315 | 0.004723425 |
| F10 | 0.9743 | 2.22907037 | 1.194004191 | 0.004627419 | 0.00999385 |
| SLC14A1 | 62.75613021 | 26.24308438 | -1.257819101 | 8.76E-11 | 2.26E-09 |
| AC018665.1 | 8.499523958 | 3.642733011 | -1.222360782 | 1.96E-14 | 2.29E-12 |
| ACSBG1 | 1.896349479 | 0.895869404 | -1.081864521 | 7.80E-10 | 1.46E-08 |
| SBSN | 25.6059875 | 110.1294274 | 2.104646916 | 0.003110267 | 0.007064972 |
| NTN1 | 3.398463542 | 7.148305636 | 1.072718674 | 6.61E-09 | 8.80E-08 |
| SLC2A3 | 15.31873646 | 36.03554589 | 1.234123397 | 1.56E-08 | 1.83E-07 |
| CD37 | 7.547628125 | 15.53451143 | 1.041381621 | 6.98E-05 | 0.000260469 |
| NID2 | 4.557904688 | 9.365531401 | 1.038990099 | 5.84E-06 | 3.04E-05 |
| ANXA1 | 194.8403776 | 431.4833802 | 1.147012307 | 1.37E-13 | 1.04E-11 |
| FBN2 | 6.126377604 | 12.58585878 | 1.038697463 | 5.10E-08 | 5.02E-07 |
| GASAL1 | 1.414265625 | 2.973081159 | 1.071905736 | 3.37E-10 | 7.16E-09 |
| AL691432.1 | 3.065813021 | 1.382406924 | -1.149087363 | 1.50E-10 | 3.62E-09 |
| CEACAM7 | 43.30406927 | 10.02217037 | -2.111307633 | 0.012297166 | 0.023320189 |
| MYH11 | 47.09453281 | 107.3852779 | 1.189164727 | 0.001990403 | 0.004788408 |
| CLSTN2 | 0.851243229 | 1.973627536 | 1.213206426 | 0.002857875 | 0.006566594 |
| AC010735.2 | 5.368994271 | 2.664151852 | -1.01097555 | 0.001787911 | 0.004357786 |
| KRT34 | 1.042651563 | 5.29328438 | 2.343906052 | 1.77E-05 | 7.87E-05 |
| CHI3L2 | 5.293941667 | 11.3118504 | 1.095420743 | 3.82E-06 | 2.09E-05 |
| CCER2 | 37.95610885 | 1.166216586 | -5.024424448 | 2.36E-10 | 5.23E-09 |
| MEX3A | 33.00124635 | 14.46053366 | -1.190399717 | 4.47E-10 | 9.06E-09 |
| TSPEAR-AS2 | 3.248268229 | 1.071961353 | -1.599417876 | 0.000944986 | 0.002489911 |
| HGF | 0.72199375 | 3.824815942 | 2.405332071 | 2.45E-08 | 2.68E-07 |
| SUGCT | 2.822200521 | 8.400266184 | 1.573614547 | 2.41E-11 | 7.57E-10 |
| FMO8P | 3.373688021 | 1.463493881 | -1.204909854 | 8.43E-06 | 4.15E-05 |
| NPR1 | 3.335349479 | 6.929462158 | 1.054905444 | 1.20E-06 | 7.70E-06 |
| TACR2 | 1.805523438 | 3.654014332 | 1.017065144 | 0.001738409 | 0.004253647 |
| AL390719.2 | 35.81278958 | 14.48866795 | -1.305549936 | 8.66E-17 | 2.67E-14 |
| AC006128.1 | 3.200248438 | 1.521935427 | -1.072276758 | 4.87E-19 | 6.11E-16 |
| WASIR2 | 3.184027604 | 1.475980193 | -1.109179482 | 6.55E-12 | 2.58E-10 |
| JAM3 | 2.378778646 | 4.769207085 | 1.003528398 | 2.44E-05 | 0.00010405 |
| GRAMD2A | 1.664427083 | 5.769819163 | 1.793500434 | 8.52E-06 | 4.18E-05 |
| CCDC80 | 12.98525833 | 29.5602839 | 1.186785409 | 1.30E-13 | 9.92E-12 |
| AP005264.1 | 1.0414375 | 2.086267794 | 1.002348093 | 8.97E-07 | 5.97E-06 |
| DPP4 | 1.390149479 | 2.841301449 | 1.031311883 | 1.74E-09 | 2.87E-08 |
| AC010487.1 | 9.268594271 | 4.198919646 | -1.142332368 | 6.22E-11 | 1.68E-09 |
| ECRG4 | 1.497473958 | 3.663159581 | 1.290557638 | 0.000843296 | 0.002259911 |
| MUC16 | 1.382829688 | 3.289272464 | 1.250145036 | 2.67E-06 | 1.53E-05 |
| ADAMTS15 | 2.583678125 | 6.242036071 | 1.272590344 | 4.60E-09 | 6.46E-08 |
| SOST | 0.656022396 | 13.4207475 | 4.354576151 | 0.0143935 | 0.026677087 |
| SNAP91 | 1.752594792 | 0.781127697 | -1.165862155 | 9.79E-06 | 4.71E-05 |
| LINC02257 | 0.833613542 | 1.923576812 | 1.206340821 | 4.16E-05 | 0.000166541 |
| TRIM31 | 52.07114271 | 19.82322705 | -1.393292227 | 0.001738411 | 0.004253647 |
| IL6 | 6.292391667 | 19.19046232 | 1.60870909 | 4.36E-08 | 4.36E-07 |
| EREG | 5.839521354 | 23.99082882 | 2.038560974 | 2.64E-09 | 4.08E-08 |
| AC034229.4 | 1.746223958 | 0.796416586 | -1.132643429 | 6.21E-13 | 3.69E-11 |
| KLK10 | 5.278059375 | 16.28622947 | 1.625573149 | 1.25E-08 | 1.51E-07 |
| EPS8L3 | 8.029842708 | 3.084982931 | -1.380109221 | 1.30E-09 | 2.25E-08 |
| MTND4P24 | 4.950347396 | 2.467502415 | -1.004478274 | 0.012239821 | 0.023228763 |
| SERPINB3 | 40.1059901 | 103.9154304 | 1.373520262 | 7.63E-06 | 3.80E-05 |
| RNASE7 | 7.111825521 | 14.63709034 | 1.041336958 | 0.009992039 | 0.019469984 |
| HOXB-AS2 | 1.913231771 | 0.811446055 | -1.237444562 | 4.66E-05 | 0.000184371 |
| ENPP1 | 0.933228646 | 4.405161514 | 2.238892419 | 1.01E-07 | 9.09E-07 |
| MFAP4 | 58.31670521 | 118.5121604 | 1.023053983 | 0.000183536 | 0.00060547 |
| CNN1 | 59.51489844 | 168.003982 | 1.497172657 | 1.81E-06 | 1.09E-05 |
| SERPINE2 | 6.438178125 | 19.29782931 | 1.583714179 | 4.96E-12 | 2.02E-10 |
| CLDN10 | 0.736050521 | 1.48427971 | 1.011886293 | 0.005754291 | 0.012083789 |
| PRR36 | 13.41768385 | 6.092589855 | -1.139008128 | 1.68E-11 | 5.68E-10 |
| AC004847.1 | 0.846538021 | 1.809969726 | 1.096318796 | 0.000312488 | 0.000957091 |
| CCDC188 | 1.938885417 | 0.93814058 | -1.047351515 | 3.73E-05 | 0.000151488 |
| AC012653.2 | 2.714367708 | 0.407801932 | -2.734675655 | 0.002711351 | 0.006272325 |
| CYP4F8 | 48.23535052 | 16.77986135 | -1.523360055 | 7.66E-15 | 1.07E-12 |
| NR4A3 | 3.667836979 | 7.561455556 | 1.043734457 | 5.04E-06 | 2.66E-05 |
| SGCA | 2.580198958 | 6.796740258 | 1.397360675 | 0.000261924 | 0.00082252 |
| AHNAK2 | 6.242006771 | 19.90677826 | 1.673177925 | 2.52E-17 | 1.13E-14 |
| IL7R | 7.224723438 | 15.65158357 | 1.115294364 | 2.56E-09 | 3.97E-08 |
| TAC1 | 2.8397875 | 1.2136781 | -1.226397147 | 0.005646709 | 0.011887893 |
| ITPKA | 2.402076563 | 5.415612077 | 1.172842266 | 0.000292746 | 0.00090485 |
| ERN2 | 16.37401042 | 7.662168438 | -1.095583072 | 5.84E-05 | 0.000223596 |
| TMEM74B | 4.036141146 | 1.769052818 | -1.189999504 | 8.35E-10 | 1.54E-08 |
| CHRDL2 | 6.022436458 | 21.8150752 | 1.856906275 | 1.11E-08 | 1.37E-07 |
| SLC22A3 | 2.658956771 | 6.44528438 | 1.277383693 | 1.44E-11 | 4.95E-10 |
| UGT2B28 | 1.592257292 | 0.489949114 | -1.700369654 | 3.75E-08 | 3.86E-07 |
| BOLA2P3 | 2.028567708 | 0.844744122 | -1.263875144 | 3.86E-08 | 3.95E-07 |
| KRT75 | 2.887179167 | 8.616613849 | 1.577460393 | 2.23E-07 | 1.80E-06 |
| ZP1 | 1.577397396 | 0.58794219 | -1.423799953 | 0.000224388 | 0.000719725 |
| SPRR1B | 171.7412505 | 614.2442929 | 1.838575946 | 0.001325705 | 0.003358084 |
| SULT1E1 | 12.99505573 | 2.7066219 | -2.26339755 | 0.000421012 | 0.001233797 |
| MYBPH | 1.661708333 | 10.42831675 | 2.649767224 | 0.002375562 | 0.005589779 |
| HOXB6 | 9.773542708 | 4.713719002 | -1.052015846 | 2.42E-06 | 1.40E-05 |
| SCEL | 3.927984896 | 13.95106795 | 1.828514277 | 1.32E-06 | 8.32E-06 |
| ECM1 | 24.42738906 | 61.71910048 | 1.337217367 | 3.53E-13 | 2.29E-11 |
| G0S2 | 48.58595521 | 111.464444 | 1.19797234 | 1.86E-07 | 1.54E-06 |
| AC019117.1 | 13.04881458 | 5.42967343 | -1.264981417 | 6.72E-07 | 4.68E-06 |
| MIR6859-1 | 3.121790104 | 1.51910789 | -1.039149203 | 1.93E-09 | 3.11E-08 |
| ATP6V0CP2 | 4.891646875 | 2.025866667 | -1.271781035 | 8.63E-06 | 4.22E-05 |
| AL355916.1 | 0.809742708 | 2.551774718 | 1.65596549 | 7.41E-05 | 0.00027486 |
| PDE10A | 4.073836979 | 1.750622705 | -1.218520063 | 3.92E-07 | 2.93E-06 |
| KCNMB1 | 2.96548125 | 6.685625443 | 1.172796281 | 5.89E-06 | 3.06E-05 |
| RHOD | 69.3612526 | 166.1419936 | 1.260214913 | 1.02E-12 | 5.44E-11 |
| PICSAR | 7.091490104 | 20.50686312 | 1.531946112 | 0.001476471 | 0.003691855 |
| AREG | 42.61135521 | 125.6462554 | 1.560057835 | 1.35E-10 | 3.30E-09 |
| STAP1 | 0.705746354 | 2.774197746 | 1.974848951 | 0.00015292 | 0.0005169 |
| PVALB | 52.79119948 | 15.30319275 | -1.786464768 | 5.97E-05 | 0.00022798 |
| GZMA | 21.45614115 | 60.20982399 | 1.488608267 | 5.77E-05 | 0.000221121 |
| MMRN1 | 1.013611979 | 2.219592754 | 1.130789518 | 0.005707646 | 0.011998222 |
| TNFRSF11B | 4.829875 | 12.60926425 | 1.38442634 | 0.000545354 | 0.001537885 |
| AC061975.6 | 2.910499479 | 0.931118357 | -1.644230289 | 0.000128682 | 0.000446056 |
| ASB5 | 0.738255208 | 1.872189855 | 1.342535209 | 5.12E-08 | 5.04E-07 |
| KRT4 | 78.1826224 | 202.0847298 | 1.37004043 | 0.001411737 | 0.003545744 |
| SFRP4 | 39.0289724 | 85.42093349 | 1.130044186 | 3.15E-06 | 1.77E-05 |
| LTO1 | 19.82877969 | 8.698310145 | -1.188788838 | 0.000345976 | 0.001042143 |
| TEKT5 | 4.446564583 | 1.878226087 | -1.243320406 | 1.18E-11 | 4.19E-10 |
| GJA5 | 4.765500521 | 12.20099597 | 1.356299267 | 2.86E-06 | 1.62E-05 |
| PSG4 | 0.930891667 | 2.756525282 | 1.566165646 | 0.021115136 | 0.03716437 |
| ITLN1 | 133.3469557 | 14.04808148 | -3.246739866 | 0.000380789 | 0.001129315 |
| AC090954.1 | 5.652684375 | 1.918259581 | -1.559138184 | 4.52E-15 | 6.89E-13 |
| AP000424.2 | 1.955804688 | 0.458739775 | -2.0920144 | 3.70E-05 | 0.000150236 |
| GFPT2 | 6.758669792 | 15.27147552 | 1.176028226 | 5.81E-11 | 1.59E-09 |
| HOXB5 | 18.02540208 | 7.84187649 | -1.200760617 | 3.80E-10 | 7.90E-09 |
| MIR4728 | 7.935805208 | 3.434547182 | -1.20825671 | 2.44E-13 | 1.68E-11 |
| AP000424.1 | 2.705317708 | 0.604695491 | -2.161517305 | 4.13E-08 | 4.16E-07 |
| B3GAT1-DT | 3.478851563 | 0.783215942 | -2.151129087 | 3.34E-11 | 1.00E-09 |
| AP000867.5 | 2.690232292 | 0.768716425 | -1.80720735 | 2.69E-11 | 8.34E-10 |
| CSF3 | 5.420507813 | 14.33005008 | 1.402543732 | 7.10E-06 | 3.59E-05 |
| SOX7 | 5.520607292 | 11.613257 | 1.072873757 | 7.04E-10 | 1.33E-08 |
| RASSF9 | 0.899035417 | 2.260105153 | 1.329940041 | 1.85E-11 | 6.12E-10 |
| F3 | 54.71455885 | 132.3662412 | 1.274538551 | 2.11E-13 | 1.48E-11 |
| AL356433.1 | 9.52494375 | 1.806857488 | -2.398227852 | 1.39E-05 | 6.38E-05 |
| AC108134.1 | 4.504388021 | 2.097806441 | -1.10244954 | 1.74E-11 | 5.84E-10 |
| FBN1 | 9.124349479 | 19.11386634 | 1.066826025 | 5.26E-11 | 1.46E-09 |
| RBP1 | 15.37281979 | 44.39217279 | 1.529923505 | 1.28E-06 | 8.09E-06 |
| CD8B | 1.434276042 | 3.755982126 | 1.388867485 | 0.008530376 | 0.017020105 |
| AL772337.3 | 4.056269271 | 1.349331401 | -1.587908704 | 2.92E-09 | 4.45E-08 |
| TUBB6 | 44.51123802 | 111.2978222 | 1.32218383 | 3.86E-17 | 1.48E-14 |
| IL1RL1 | 0.884195313 | 2.24967037 | 1.347276637 | 5.16E-05 | 0.000201047 |
| LAMA1 | 1.106000521 | 2.228789855 | 1.010908531 | 1.25E-07 | 1.09E-06 |
| GPX3 | 51.89403229 | 159.6215167 | 1.621014592 | 8.91E-09 | 1.14E-07 |
| MUC4 | 6.071948438 | 12.57957391 | 1.050851612 | 0.009207311 | 0.01816649 |
| CRISPLD2 | 16.200725 | 33.02689903 | 1.027583142 | 5.59E-08 | 5.45E-07 |
| ACKR1 | 12.80330156 | 35.35459968 | 1.465382041 | 0.012930933 | 0.024319817 |
| GJB2 | 239.073351 | 482.6720155 | 1.013589859 | 6.80E-05 | 0.000254939 |
| TDH | 2.761165104 | 1.298467472 | -1.088467283 | 4.08E-10 | 8.37E-09 |
| TRPS1 | 2.355080729 | 4.767155878 | 1.017352285 | 1.61E-13 | 1.20E-11 |
| IL1RAP | 4.9731375 | 10.04588357 | 1.014376235 | 1.29E-10 | 3.18E-09 |
| BCHE | 1.344904167 | 4.52945475 | 1.751834016 | 4.88E-09 | 6.81E-08 |
| LAIR2 | 0.952501042 | 2.056966989 | 1.110726065 | 0.002926774 | 0.00670132 |
| KLK6 | 7.394041667 | 36.80553366 | 2.315487611 | 6.19E-08 | 5.97E-07 |
| TNFAIP6 | 7.478103646 | 15.4770905 | 1.049389916 | 5.48E-10 | 1.08E-08 |
| AL158175.1 | 5.825220313 | 1.517297424 | -1.940808701 | 2.49E-07 | 1.97E-06 |
| CRTAC1 | 64.10549323 | 22.25037778 | -1.526618156 | 2.23E-07 | 1.80E-06 |
| CD48 | 2.345486979 | 4.693709823 | 1.000841162 | 3.71E-05 | 0.000150747 |
| CAVIN1 | 152.3015 | 311.4461878 | 1.032052762 | 1.22E-15 | 2.22E-13 |
| PAX5 | 0.617555729 | 2.232228502 | 1.853843478 | 0.000383366 | 0.001136314 |
| EMX2 | 13.07054583 | 4.831822222 | -1.435680111 | 5.01E-08 | 4.94E-07 |
| DSP | 130.7530953 | 262.1096335 | 1.003325279 | 7.02E-08 | 6.64E-07 |
| FOSL1 | 43.97093698 | 93.61494364 | 1.090188568 | 1.43E-08 | 1.70E-07 |
| AC040174.2 | 1.909175 | 0.847388567 | -1.171853781 | 4.79E-05 | 0.00018852 |
| AC003070.1 | 5.136633854 | 2.513967794 | -1.030857073 | 6.68E-14 | 5.89E-12 |
| CLC | 0.734389583 | 1.945735266 | 1.405697932 | 0.006275918 | 0.013054902 |
| TLL1 | 0.690430208 | 2.052692915 | 1.571950322 | 6.45E-09 | 8.63E-08 |
| CD180 | 1.289463021 | 2.875136554 | 1.156860078 | 4.89E-05 | 0.00019197 |
| RNU6-50P | 1.896434896 | 0.91943124 | -1.044476254 | 1.59E-08 | 1.86E-07 |
| BX571818.1 | 13.3545724 | 4.665798873 | -1.517137759 | 0.002248629 | 0.005324104 |
| LINC02598 | 2.932417188 | 1.059042834 | -1.469329425 | 3.93E-12 | 1.67E-10 |
| CYP4F12 | 35.06422813 | 11.3935467 | -1.621783056 | 1.98E-14 | 2.29E-12 |
| CCL20 | 18.1292375 | 44.40862786 | 1.292521748 | 0.010547534 | 0.020437725 |
| ALB | 14.12086302 | 2.807808374 | -2.330311881 | 0.025809598 | 0.044031153 |
| THBS1 | 64.62249844 | 147.7459924 | 1.193010564 | 1.27E-13 | 9.88E-12 |
| MMP9 | 30.07159792 | 172.8249573 | 2.522838134 | 1.36E-07 | 1.17E-06 |
| SH3GL3 | 0.618253125 | 1.425839775 | 1.205542339 | 4.98E-07 | 3.62E-06 |
| COMP | 42.38687656 | 111.8594847 | 1.399998024 | 1.89E-05 | 8.32E-05 |
| NXPH3 | 1.6226125 | 3.275806602 | 1.013531678 | 0.001004314 | 0.002622949 |
| CRABP1 | 3.011130729 | 0.96407649 | -1.643085825 | 0.000591461 | 0.001649763 |
| CSDC2 | 2.873679688 | 6.102378744 | 1.086472462 | 4.27E-07 | 3.16E-06 |
| CYP2C9 | 2.64700625 | 0.764507246 | -1.79175952 | 0.000165819 | 0.000553964 |
| CLCF1 | 13.22774115 | 28.10355813 | 1.08718608 | 4.59E-09 | 6.45E-08 |
| DIRAS3 | 1.2649375 | 2.906837359 | 1.20038425 | 7.91E-05 | 0.00029106 |
| COL21A1 | 0.886447396 | 2.33909211 | 1.399841748 | 0.001453777 | 0.003639583 |
| SHISAL1 | 1.117086458 | 2.241738647 | 1.004877242 | 1.34E-07 | 1.15E-06 |
| RGS2 | 42.70119115 | 92.51838631 | 1.115463789 | 3.41E-12 | 1.47E-10 |
| CTSV | 8.65599375 | 22.65508889 | 1.388063789 | 2.41E-07 | 1.92E-06 |
