# Supplementary material for: Identification and validation of a novel cuproptosis-related lncRNA gene signature to predict prognosis and immune response in bladder cancer
Source: Discov Oncol. 2022 Dec 1;13:133. doi: 10.1007/s12672-022-00596-w (PMC9715909; doi:10.1007/s12672-022-00596-w)
Supplement: Supplementary file 2 — Additional file2 (DOCX 11 KB) [file 12672_2022_596_MOESM2_ESM.docx]

| **cuprotosis-related genes** |
| --- |
| NFE2L2 |
| NLRP3 |
| ATP7B |
| ATP7A |
| SLC31A1 |
| FDX1 |
| LIAS |
| LIPT1 |
| LIPT2 |
| DLD |
| DLAT |
| PDHA1 |
| PDHB |
| MTF1 |
| GLS |
| CDKN2A |
| DBT |
| GCSH |
| DLST |

**Table S2: Cuprotosis-related Genes**
